# SIMUnet: an open-source tool for simultaneous global fits of EFT Wilson coefficients and PDFs

**DOI:** 10.1140/epjc/s10052-024-13079-9

**Published:** 2024-08-12

**Authors:** PBSP Collaboration, Mark N. Costantini, Elie Hammou, Zahari Kassabov, Maeve Madigan, Luca Mantani, Manuel Morales-Alvarado, James M. Moore, Maria Ubiali

**Affiliations:** 1 DAMTP, University of Cambridge, Wilberforce Road, Cambridge, CB3 0WA UK; 2 Institut für Theoretische Physik, Universität Heidelberg, Philosophenweg 16, D-69120 Heidelberg, Germany

## Abstract

We present the open-source SIMUnet code, designed to fit Standard Model Effective Field Theory (SMEFT) Wilson coefficient alongside Parton Distribution Functions (PDFs) of the proton. SIMUnet can perform SMEFT global fits, as well as simultaneous fits of the PDFs and of an arbitrarily large number of SMEFT degrees of freedom, by including both PDF-dependent and PDF-independent observables. SIMUnet can also be used to determine whether the effects of any New Physics models can be fitted away in a global fit of PDFs. SIMUnet is built upon the open-source NNPDF code and is released together with documentation, and tutorials. To illustrate the functionalities of the new tool, we present a new global analysis of the SMEFT Wilson coefficients accounting for their interplay with the PDFs. We increment our previous analysis of the LHC Run II top quark data with both (i) the Higgs production and decay rates data from the LHC, and (ii) the precision electroweak and diboson measurements from LEP and the LHC.

## Introduction

The success of the ambitious programme of the upcoming Run III at the LHC and its subsequent High-Luminosity (HL-LHC) upgrade [1, 2] relies not only on achieving the highest possible accuracy in the experimental measurements and in the corresponding theoretical predictions, but also on the availability of statistically robust tools capable of yielding global interpretations of all subtle deviations from the Standard Model (SM) that the data might indicate. The lack of evidence for additional particles at the LHC or at other colliders so far suggests that any particles beyond the Standard Model (BSM) may be significantly heavier than the scale probed by the LHC. Hence, the effects of heavy BSM particles may be approximated by integrating them out to obtain higher-dimensional interactions between the SM fields [3]. The SM may then be seen as an effective field theory (EFT) and can be supplemented by higher-dimensional operators that are suppressed by inverse powers of new particles’ mass scale [4–6].

The Standard Model Effective Field Theory (SMEFT) is a powerful framework to constrain, identify, and parametrise potential deviations with respect to SM predictions; see Ref. [7] for a review. It allows for the interpretation of experimental measurements in the context of BSM scenarios featuring heavy new particles while minimising assumptions on the nature of the underlying UV-complete theory. Early SMEFT analyses constrained subsets of SMEFT operators relevant to sectors of observables, for example the electroweak, diboson and Higgs sectors [8–16], the top sector [17–22] as well as Drell–Yan [23, 24]. More recently, global fits constraining a larger set of SMEFT operators have been performed: the Higgs, top, diboson and electroweak sectors have been combined [25–27], as well as subsets of those [28] along with low-energy charged-current observables [29, 30]. Also, a combinations of all those sectors alongside Drell–Yan and flavour observables has been recently published [31].

The determination of SMEFT Wilson coefficients from a fit of LHC data, like the determination of SM precision parameters from LHC data, might display a non-negligible interplay with the input set of Parton Distribution Functions (PDFs) used to compute theory predictions. This was shown in several recent studies [21, 32–36], in which such interplay between PDFs and SMEFT Wilson coefficients was quantified for the first time in relevant phenomenological scenarios. For example, in [32] it was shown that, while the effect of four-fermion operators on Deep Inelastic Scattering (DIS) data can be non-negligible, if DIS data were fitted while taking the effect of such operators into account, the fit quality would deteriorate proportionally to the energy scale $Q^2$ of the data included in the determination. On the other hand in the context of high-mass Drell–Yan, especially in the HL-LHC scenario, neglecting the cross-talk between the large-*x* PDFs and the SMEFT effects in the tails could potentially miss new physics manifestations or misinterpret them [33, 36, 37], as the bounds on SMEFT operators are significantly broader if the PDFs are fitted by including the effect of universal operators in the high energy tails of the data. In [36] it was shown that the large-*x* antiquark distributions can absorb the effect of universal new physics in the tails of the Drell–Yan distributions by leading to significantly softer antiquark distributions in the large-*x* region, far beyond the nominal PDF uncertainties. The analysis of the top quark sector of Ref. [21] demonstrates that in that case the bounds of the operators are not broadened by the interplay with the PDFs, however the correlation between the top sector and the gluon is manifest in the gluon PDF itself, which becomes softer in the large-*x* region if the PDF fit is augmented by the top data and PDFs are fitted simultaneously along the Wilson coefficients that determine the top quark sector.

The first exploration of how the Wilson coefficients of the SMEFT and the proton PDFs are intertwined [32–34] was somewhat limited by methodologies that allowed for the consideration of only a handful of SMEFT operators, as they were based on a scan in the operator space. SIMUnet was the first tool that allowed for a truly simultaneous fit of the PDFs alongside any physical parameter that enters theoretical predictions, whether a precision SM parameter, or the Wilson coefficients of a SMEFT expansion without any hard limits in the number of parameters that are fitted alongside the PDFs. The new framework was first presented in Ref. [35] in the context of high-mass Drell–Yan distributions. Later in Ref. [21] it was applied to analyse the broadest top quark dataset used to date in either PDF or SMEFT interpretations, which in particular contained all available measurements in the top sector from the ATLAS and CMS experiments based on the full Run II luminosity. By combining this wide dataset the potentiality of the SIMUnet methodology was showcased by using it to derive bounds on 25 independent Wilson coefficients, both independently and alongside the PDFs.

In this paper, we present and publicly release the computational framework SIMUnet, so that any user can assess the interplay between PDFs and New Physics, either by performing simultaneous fits of SMEFT operators (or operators in any other EFT expansion) alongside the PDFs of the proton, or by injecting any New Physics model in the data and checking whether a global fit of PDFs can absorb the effects induced by such a model in the data.

SIMUnet is based on the first tagged version of the public NNPDF code [38, 39] associated with the NNPDF4.0 PDF set release [40–42], which it augments with a number of new features that allow the exploration of the correlation between a fit of PDFs and BSM degrees of freedom. First of all, it allows for the simultaneous determination of PDFs alongside any Wilson coefficients of an EFT that enters in the theory predictions. The user can specify any subset of operators that are of phenomenological relevance, compute the corresponding corrections to the SM predictions, and derive bounds on the operators from all observables entering the PDF fit and an arbitrary number of PDF-independent observables that can be added on top of the PDF-dependent ones. Histograms of the bounds on Wilson coefficients, correlation coefficients between PDF and Wilson coefficients, as well as Fisher information matrices and bound comparisons are automatically produced by the validphys analysis framework, a package implemented in reportengine [43] that allows for the analysis and plotting of data related to the SIMUnet fit structures and input/output capabilities to other elements of the code base. Users can also inject the effects of any New Physics scenario in the data, and assess whether PDFs might absorb them and fit them away, in the context of a closure test based on artificial data, as carried out in Ref. [36].

Furthermore, we present a new global simultaneous analysis of SMEFT Wilson coefficients and PDFs based on a broad range of observables, including for the first time (i) the Higgs production and decay rates data from the LHC, (ii) precision electroweak and diboson measurements from LEP and the LHC, and (iii) Drell–Yan measurements, in a global analysis of 40 dimension-6 SMEFT operators included linearly. The Wilson coefficients are determined both with fixed PDFs and simultaneously with the PDFs themselves.

The structure of the paper is the following. In Sect. 2 we review the SIMUnet methodology and describe in detail how it is implemented in the code. This section is especially relevant for those who wish to use the public code to perform simultaneous fits of PDFs and Wilson coefficients of an EFT. In Sect. 3 we describe the use of the code in assessing whether New Physics signals can be absorbed by PDFs.

In Sect. 4 we present all new data implemented for this release, and present the results we obtain in the first global SMEFT analysis that explores up to 40 Wilson Coefficients and fits them alongside PDFs. Before concluding and providing the link to the public SIMUnet repository and outlining the plan for future developments in Sect. 6, in Sect. 5 we summarise tips for users on the applicability of the code, its limitations and its proper usage and provide the links to the public code repository and to the online documentation.

## SIMUnet: methodology for simultaneous fits and code structure

In this section, we provide a broad overview of the general methodology and code structure of SIMUnet, in particular describing its functionality regarding simultaneous PDF-SMEFT fits. In Sect. 2.1 we begin by briefly reviewing the NNPDF4.0 framework and code, which SIMUnet builds upon, and then in Sect. 2.2 we review the specific SIMUnet methodology and code implementation. In Sect. 2.3 we give some details of usage (in particular the user-facing yaml run card structure), but the user is encouraged to check the supporting webpage, https://hep-pbsp.github.io/SIMUnet/, for a more comprehensive description.

### Review of the NNPDF4.0 framework

The central premise of the NNPDF4.0 methodology [39, 40] is the *neural network* parametrisation of the PDFs of the proton. The NNPDF4.0 methodology assumes that one can write the eight fitted PDF flavours at the parametrisation scale $Q_0^2$, ${\textbf {f}}(x, Q_0^2) \in {\mathbb {R}}^8$, in the form^1^:

2.1 $$\begin{aligned} {\textbf {f}}(x,Q_0^2) = {\textbf {NN}}(x, \pmb {\omega }), \end{aligned}$$

where ${\textbf {NN}}(\cdot ,\pmb {\omega }): [0,1] \rightarrow {\mathbb {R}}^8$ denotes a suitable neural network, and $\pmb {\omega }$ are the parameters of the network. Given an $N_{\text {dat}}$-dimensional dataset ${\textbf {D}} \in {\mathbb {R}}^{N_{\text {dat}}}$, the corresponding theory predictions ${\textbf {T}}(\pmb {\omega },{\textbf {c}}) \in {\mathbb {R}}^{N_{\text {dat}}}$ are constructed from this neural network parametrisation via the following discretisation of the standard collinear factorisation formula:

2.2 $$\begin{aligned} T_i(\pmb {\omega },{\textbf {c}}) = {\left\{ \begin{array}{ll} \displaystyle \sum _{a = 1}^{N_{\text {flav}}} \sum _{\alpha =1}^{N_{\text {grid},i}} \text {FK}_{i,a\alpha }({\textbf {c}}) \text {NN}_a(x_\alpha ,\pmb {\omega }), &{} \text {if}\ D_i \ \text {is deep-inelastic scattering data;} \\ \displaystyle \sum _{a,b=1}^{N_{\text {flav}}} \sum _{\alpha ,\beta =1}^{N_{\text {grid},i}} \text {FK}_{i,a\alpha b\beta }({\textbf {c}}) \text {NN}_a(x_\alpha ,\pmb {\omega }) \text {NN}_b(x_\beta ,\pmb {\omega }), &{} \text {if} \ D_i \ \text {is hadronic data.}\end{array}\right. } \end{aligned}$$

Here, $\text {FK}_{i,a\alpha }({\textbf {c}})$ (or $\text {FK}_{i,a\alpha b \beta }({\textbf {c}})$) is called the *fast-kernel (FK) table* for the *i*th datapoint, which encompasses both the partonic cross section for the process associated to the *i*th datapoint and the coupled evolution of the PDFs from the parametrisation scale $Q_0^2$ to the scale $Q^2_i$ associated to the datapoint *i*, and $(x_1,...,x_{N_{\text {grid},i}})$ is a discrete *x*-grid for the *i*th datapoint. Note importantly that the theory predictions carry a dependence on the parameters of the neural network, $\pmb {\omega }$, but additionally on a vector of physical constants ${\textbf {c}} \in {\mathbb {R}}^{N_{\text {param}}}$ through the FK-tables, such as the strong coupling $\alpha _s(M_z)$, the electroweak parameters in a given electroweak input scheme, the CKM matrix elements, or the masses of the heavy quarks, which are fixed to some reference values for any given NNPDF4.0 fit. A choice of constants ${\textbf {c}}$ determines a corresponding set of FK-tables, which is referred to as a ‘theory’ in the NNPDF4.0 parlance.

A PDF fit in the NNPDF4.0 framework comprises the determination of the neural network parameters $\pmb {\omega }$, given a choice of theory ${\textbf {c}} = {\textbf {c}}^*$, together with an uncertainty estimate for these parameters. This is achieved via the *Monte Carlo replica method*, described as follows. Suppose that we are given experimental data ${\textbf {D}}$, together with a covariance matrix $\Sigma$. We generate $N_{\text {rep}}$ ‘pseudodata’ vectors, ${\textbf {D}}_1,..., {\textbf {D}}_{N_{\text {rep}}}$, as samples from the multivariate normal distribution:

2.3 $$\begin{aligned} {\textbf {D}}_i \sim {\mathcal {N}}({\textbf {D}}, \Sigma ). \end{aligned}$$

The default choice for the covariance matrix $\Sigma$ is $\Sigma = \Sigma _{\textrm{exp}}$, i.e. the experimental covariance matrix, which includes all information on experimental uncertainties and correlations. The latter can also be augmented by a theory covariance matrix $\Sigma _{\textrm{th}}$, hence setting $\Sigma = \Sigma _{\textrm{exp}} + \Sigma _{\textrm{th}}$, where $\Sigma _{\textrm{th}}$ includes the effects of nuclear correction uncertainties [45, 46] and missing higher order uncertainties [41, 47, 48] in the theory predictions used in a PDF fit. An alternative approach to keep account of theory uncertainties in the fit is to expand the Monte Carlo sampling to the space of factorisation and renormalisation scale parameters; this approach was presented in Ref. [49]. Both options can be implemented in the SIMUnet framework, the former in a straightforward way, the latter with some modifications that we leave to future releases.

For each pseudodata ${\textbf {D}}_k$, we find the corresponding best-fit PDF parameters $\pmb {\omega }_k$ by minimising the $\chi _k^2$ loss function, defined as a function of $\pmb {\omega }$ as

2.4 $$\begin{aligned} \chi ^2_k(\pmb {\omega }, {\textbf {c}}^*) = ({\textbf {D}}_k - {\textbf {T}}(\pmb {\omega }, {\textbf {c}}^*))^T \Sigma _{t_0} ({\textbf {D}}_k - {\textbf {T}}(\pmb {\omega }, {\textbf {c}}^*)) \,, \end{aligned}$$

in which the $t_0$ covariance matrix (rather than the experimental covariance matrix $\Sigma _{\textrm{exp}}$) is used. The use of the $t_0$ covariance matrix is designed to avoid the so-called D’Agostini bias [50] and ensure a faithful propagation of multiplicative uncertainties, as described in Ref. [51]. The latter is defined as

2.5 $$\begin{aligned} \left( \Sigma _{t_0 }\right) _{i j}= & {} \delta _{i j}\sigma _i^{\text{(uncorr) } }\sigma _j^{\text{(uncorr) } }\nonumber \\{} & {} +\sum _{m=1}^{N_{\textrm{norm}}} \sigma _{i, m}^{(\mathrm norm)} \sigma _{j, m}^{(\mathrm norm)}T_i^{(0)} T_j^{(0)}\nonumber \\{} & {} +\sum _{l=1}^{N_{\textrm{corr}}} \sigma _{i, l}^{(\mathrm corr)} \sigma _{j, l}^{(\mathrm corr)}D_i D_j, \end{aligned}$$

where $T^{(0)}_i$ is a theoretical prediction for the *i*-th data point evaluated using a $t_0$ input PDF. This leads to a set of PDFs that is then used to iteratively compute the theoretical predictions $T_i^{(0)}$ that are needed for the construction of a new $t_0$ covariance matrix, which is then used for a new PDF determination. This procedure is iterated until convergence. The minimisation is achieved using *stochastic gradient descent*, which can be applied here because the analytic dependence of $\chi ^2_k(\pmb {\omega }, {\textbf {c}}^*)$ on $\pmb {\omega }$ is known, since the neural network parametrisation is constructed from basic analytic functions as building blocks. Furthermore, the $\chi ^2$-loss is additionally supplemented by *positivity* and *integrability* penalty terms; these ensure the positivity of observables and the integrability of the PDFs in the small-*x* region.

Given the best-fit parameters $\pmb {\omega }_1,..., \pmb {\omega }_{N_{\text {rep}}}$ to each of the pseudodata, we now have an ensemble of neural networks which determine an ensemble of PDFs, ${\textbf {f}}_1,..., {\textbf {f}}_{N_{\text {rep}}}$, from which statistical estimators can be evaluated, such as the *mean* or *variance* of the initial-scale PDFs.

*Review of the* NNPDF4.0 *code.* The methodology sketched above is at the basis of the NNPDF public code [38]. The code comprises several packages. To transform the original measurements provided by the experimental collaborations, e.g. via HepData [52], into a standard format that is tailored for fitting, the NNPDF code uses the buildmaster C++ experimental data formatter. Physical observables are evaluated as a tensor sum as in Eq. (2.2) via the APFELcomb [53] package that takes hard-scattering partonic matrix element interpolators from APPLgrid [54] and FastNLO [55] (for hadronic processes) and apfel[56] (for DIS structure functions) and combines them with the QCD evolution kernels that evolve the initial-scale PDFs. The package also handles NNLO QCD and/or NLO electroweak *K*-factors when needed. The actual fitting code is implemented in the TensorFlow framework [57] via the n3fit library. The latter allows for a flexible specification of the neural network model adopted to parametrise the PDFs, whose settings can be selected automatically via the built-in hyper-optimisation tooling [58], such as the neural network architecture, the activation functions, and the initialisation strategy; the choice of optimiser and of the hyperparameters related to the implementation in the fit of theoretical constraints such as PDF positivity [59] and integrability. Finally the code comprises the validphys analysis framework, which analyses and plots data related to the NNPDF fit structures, and governs input/output capabilities of other elements of the code base.^2^

### The SIMUnet framework

An important aspect of the NNPDF4.0 methodology, as well as of most global PDF analyses, is that the physical parameters ${\textbf {c}}$ must be chosen and fixed before a PDF fit, so that the resulting PDFs are produced *under the assumption* of a given theory ${\textbf {c}} = {\textbf {c}}^*$. It is desirable (and in fact necessary in some scenarios; see for example Sect. 5.3 of Ref. [33]) to relax this requirement, and instead to fit both the PDFs *and* the physical parameters ${\textbf {c}}$ *simultaneously*.

On regarding Eq. (2.4), one may assume that this problem can immediately be solved by applying stochastic gradient descent not only to the parameters of the neural network $\pmb {\omega }$, but to the tuple $(\pmb {\omega }, {\textbf {c}})$, without first fixing a reference value ${\textbf {c}} = {\textbf {c}}^*$. Unfortunately, this is not a solution in practice; the FK-tables typically have an extremely complex, non-analytical, dependence on the physical parameters ${\textbf {c}}$, and evaluation of a complete collection of FK-tables at even one point ${\textbf {c}} = {\textbf {c}}^*$ requires hundreds of computational hours and an extensive suite of codes. Hence, since the dependence of the FK-tables as an analytic function of ${\textbf {c}}$ is unavailable, it follows that ${\textbf {T}}(\pmb {\omega },{\textbf {c}})$ is not a known analytic function of ${\textbf {c}}$, and stochastic gradient descent *cannot* be applied.

Thus, if we would like to continue with our programme of simultaneous extraction of PDFs and physical parameters, we will need to approximate. The central conceit of the SIMUnet methodology is the observation that for many phenomenologically-interesting parameters, particularly the Wilson coefficients of the SMEFT expansion, the dependence of the theory predictions ${\textbf {T}}(\pmb {\omega }, {\textbf {c}})$ on the physical parameters ${\textbf {c}}$ can be well-approximated using the linear ansatz^3^

2.6 $$\begin{aligned} {\textbf {T}}(\pmb {\omega }, {\textbf {c}}) \approx {\textbf {T}}(\pmb {\omega }, {\textbf {c}}^*) \odot \left[ {\textbf {1}} + {\textbf {K}}_{\text {fac}}(\pmb {\omega }^*) ({\textbf {c}} - {\textbf {c}}^*) \right] , \end{aligned}$$

where $\odot$ denotes the element-wise Hadamard product of vectors,^4^${\textbf {1}} \in {\mathbb {R}}^{N_{\text {dat}}}$ is a vector of ones, ${\textbf {c}}^*$ is some reference value of the physical parameters, and ${\textbf {K}}_{\text {fac}}(\pmb {\omega }^*) \in {\mathbb {R}}^{N_{\text {dat}} \times N_{\text {param}}}$ is a matrix of pre-computed ‘*K*-factors’:

2.7 $$\begin{aligned}{} & {} K_{\text {fac}}(\pmb {\omega }^*)_{ij}\nonumber \\{} & {} \quad = \frac{T_i(\pmb {\omega }^*, c_1^*,..., c_{j-1}^*,c_j^* + 1,c_{j+1}^*,..., c_{N_{\text {param}}}^*) - T_i(\pmb {\omega }^*,{\textbf {c}}^*)}{T_i(\pmb {\omega }^*, {\textbf {c}}^*)},\nonumber \\ \end{aligned}$$

which are designed to approximate the appropriate (normalised) gradients:

2.8 $$\begin{aligned} K_{\text {fac}}(\pmb {\omega }^*)_{ij} \approx \frac{1}{T_i(\pmb {\omega }^*, {\textbf {c}}^*)} \frac{\partial T_i}{\partial c_j}(\pmb {\omega }^*, {\textbf {c}}^*). \end{aligned}$$

Observe that the *K*-factors are determined with a fixed choice of reference PDF, $\pmb {\omega } = \pmb {\omega }^*$, where they should technically depend on PDFs freely. In practice however, this approximation is often justified, and the reliability of the approximation can always be checked post-fit; see Appendix C of Ref. [33] for an example of this validation.

Note that the dependence of the theory in Eq. (2.6) on $\pmb {\omega }$ *and* ${\textbf {c}}$ is now known as an analytic function. The SIMUnet methodology, at its heart, now simply extends the NNPDF4.0 methodology by replacing the theory predictions in Eq. (2.4) with those specified by Eq. (2.6), and then running stochastic gradient descent on a series of Monte Carlo pseudodata as in the NNPDF4.0 framework. The result of the fit is a series of best-fit tuples $(\pmb {\omega }_1, {\textbf {c}}_1)$,..., $(\pmb {\omega }_{N_{\text {rep}}}, {\textbf {c}}_{N_{\text {rep}}})$ to the various pseudodata, from which statistical estimators can be calculated as above.

### Structure and usage of the SIMUnet code

**Fig. 1:**
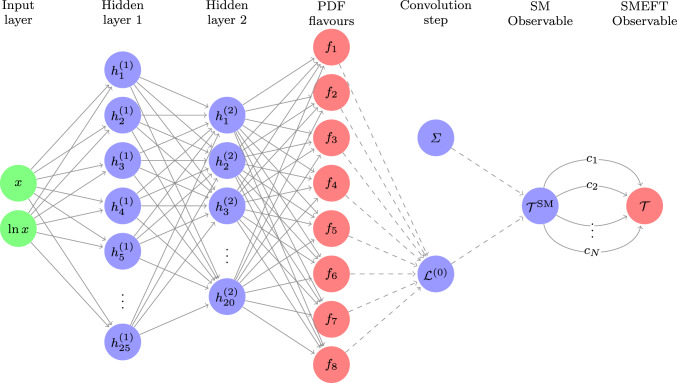
A schematic representation of the neural network parametrisation of ${\textbf {T}}(\pmb {\omega }, {\textbf {c}})$ used by the SIMUnet code. The usual NNPDF network is represented by the layers from the ‘Input layer’ to the ‘SM Observable’ layer. The final ‘SMEFT observable’ layer is related to the ‘SM observable’ layer by edges which carry the theory parameters ${\textbf {c}}$ as weights

The SIMUnet code is a fork of the NNPDF4.0 public code [39], where the key modification is the replacement of the standard theory predictions used in the $\chi ^2$-loss, Eq. (2.4), by NNPDF4.0 with the approximate formula for the theory predictions given in Eq. (2.6). This replacement is effected by the inclusion of a new post-observable *combination layer* in the network, specified by the CombineCfac.py layer added to the n3fit/layers directory; see Fig. 1 for a schematic representation.

Beyond the inclusion of this layer, the main modification to the NNPDF4.0 code comprises reading of the *K*-factors required in the approximation shown in Eq. (2.6). The *K*-factors are implemented in a new file format, the simu_fac format. One simu_fac file is made available for each dataset. The simu_fac files are packaged inside the relevant NNPDF4.0 theory folders, inside the simu_factors directory; an example of the correct structure is given inside theory_270, which is a theory available as a separate download to the SIMUnet release. As an example of the files, consider the file SIMU_ATLAS_CMS_WHEL_8TEV.yaml from the directory theory_270/simu_factors:
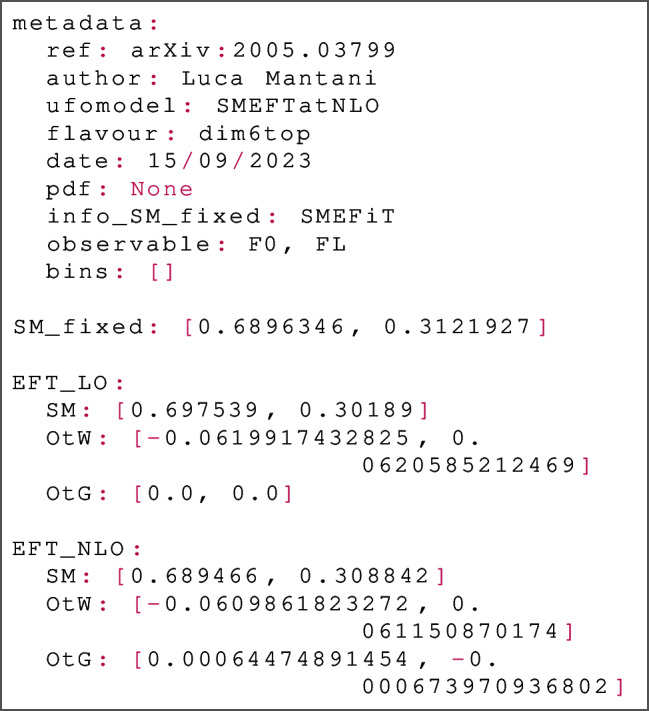


The structure of the simu_fac file is as follows:

- There is a metadata namespace at the beginning of the file, containing information on the file. The metadata is never read by the code, and is only included as a convenience to the user.
- The SM_fixed namespace provides the best available SM predictions; these may be calculated at next-to-leading order or next-to-next-to-leading order in QCD, including or excluding EW corrections, depending on the process.
- The remaining parts of the file contain the information used to construct *K*-factors for different models. In this file, two models are included: the SMEFT at leading order in QCD, and the SMEFT at next-to-leading order in QCD.

In order to perform a simultaneous fit of PDFs and the parameters specified in a particular model of a simu_fac file, we must create a run card. The runcard follows the standard NNPDF4.0 format, details and examples of which are given on the website https://hep-pbsp.github.io/SIMUnet/. We provide here a discussion of the modifications necessary for a SIMUnet fit. First, we must modify the dataset_inputs configuration key. Here is an example of an appropriate dataset_inputs for a simultaneous fit of PDFs and SMEFT Wilson coefficients at next-to-leading order in QCD:
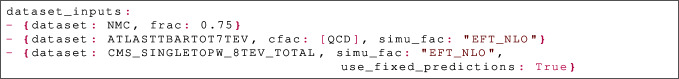


Here, we are fitting three datasets: the fixed-target DIS data from the New Muon Collaboration [60, 61] (NMC), the total $t{\bar{t}}$ cross section measured by ATLAS at $\sqrt{s}=7$ TeV [62] (ATLASTTBARTOT7TEV) and the total associated single top and *W* boson cross section measure by CMS at $\sqrt{s}=8$ TeV [63] (CMS_SINGLETOPW_8TEV_TOTAL), which we discuss in turn:

1. First, note that the dataset NMC is entered exactly as it would appear in an NNPDF4.0 runcard; it is therefore treated by SIMUnet as a standard dataset for which there is no *K*-factor modification, i.e. it depends purely on the PDFs and no additional physics parameters.
2. On the other hand, the dataset ATLASTTBARTOT7TEV has an additional key included, namely simu_fac, set to the value EFT_NLO; this tells SIMUnet to treat theory predictions for this dataset using the approximation Eq. (2.6), with the *K*-factors constructed from the EFT_NLO model specified in the relevant simu_fac file. Precisely *which* parameters from the model are fitted is determined by the simu_parameters configuration key, which we describe below.
3. Finally, observe that the dataset CMS_SINGLETOPW_8TEV_TOTAL includes two new keys: simu_fac and use_fixed_predictions. The first key, simu_fac has exactly the same interpretation as with the previous dataset; on the other hand, the fact that the key use_fixed_predictions is set to True instead removes the PDF-dependence of this dataset. That is, the predictions for this dataset are simplified even from Eq. (2.6), to:
  2.9 $$\begin{aligned} {\textbf {T}}(\pmb {\omega }, {\textbf {c}}) \approx {\textbf {T}}_{\text {fixed}} \odot \left[ {\textbf {1}} + {\textbf {K}}_{\text {fac}}(\pmb {\omega }^*) ({\textbf {c}} - {\textbf {c}}^*) \right] , \end{aligned}$$
  where ${\textbf {T}}_{\text {fixed}}$ is the vector of predictions taken from the SM_fixed namespace of the simu_fac file. Note that the right hand side no longer has a dependence on $\pmb {\omega }$, so this dataset effectively becomes PDF-independent. This is appropriate for observables which do not depend on the PDFs (e.g. the electroweak coupling, or *W*-helicities in top decays), but also observables which depend only weakly on the PDFs and for which the full computation of FK-tables would be computationally expensive.

Outside of the dataset_inputs configuration key, we must also include a new key, called simu_parameters. This specifies *which* of the parameters in the model read from the simu_fac files will be fitted, and additionally specifies hyperparameters relevant to their fit. An example is:
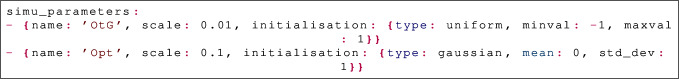


This specification tells SIMUnet that for each of the datasets which have set a model value for simu_fac, the relevant model in the simu_fac file should be checked for the existence of each of the parameters, and their contribution should be included if they appear in the model in the file. Observe the following:

1. The scale can be used to modify the learning rate in the direction of each of the specified parameters. In detail, suppose that the scale $\lambda$ is chosen for the parameter *c*. Then, SIMUnet multiplies the relevant *K*-factor contribution by $1/\lambda$ so that we are effectively fitting the parameter $\lambda c$ instead of *c* itself. When *K*-factor contributions from parameters are particularly large, setting a large scale can improve training of the network, avoiding exploring parts of the parameter space which have extremely poor $\chi ^2$s; see Ref. [35] for further discussion. On the other hand, setting a small scale can speed up training of the network by increasing the effective step size in a particular direction in parameter space; the user must tune these scales by hand to obtain optimal results. An automatic scale choice feature may be included in a future release.
2. The initialisation key informs SIMUnet how to initialise the parameters when training commences; this initialisation is random and there are three basic types available. If uniform is selected, the initial value of the parameter is drawn from a random uniform distribution between the keys minval and maxval, which must be additionally specified. If gaussian is selected, the initial value of the parameter is drawn from a random Gaussian distribution with mean and standard deviation given by the keys mean and std_dev respectively. Finally, if constant is chosen, the key value is supplied and the initial value of the parameter is always set to this key (no random selection is made in this instance).

It is also possible to specify *linear combinations* of the model parameters to fit; this feature is useful because the supplied simu_fac files only contain SMEFT models with Wilson coefficients in the *Warsaw basis* [6]. For example, the user may want to fit in a different basis to the Warsaw basis, or may wish to fit a UV model which has been matched to the SMEFT at dimension 6, with parameters given by combinations of the SMEFT parameters. An example is the following:
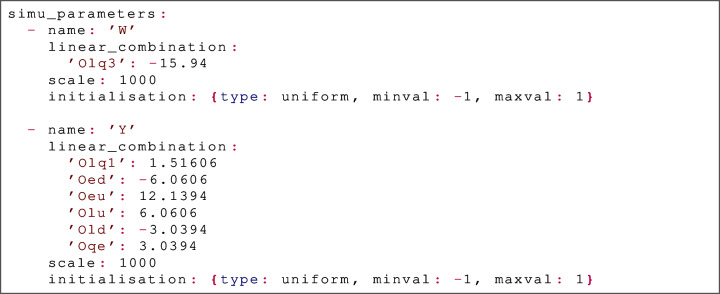


using the ${\hat{W}}$ and ${\hat{Y}}$ parameters studied in Ref. [33]. Here, we fit the user-defined operators:

$$\begin{aligned} {\hat{W}}&= -15.94 \;{\mathcal {O}}_{lq}^{(3)}, \\ {\hat{Y}}&= 1.51606 \; {\mathcal {O}}_{lq}^{(1)} - 6.0606 \; {\mathcal {O}}_{ed} + 12.1394 \; {\mathcal {O}}_{eu}\\&\quad + 6.0606 \; {\mathcal {O}}_{lu} - 3.0394 \; {\mathcal {O}}_{ld} + 3.0394 \; {\mathcal {O}}_{qe}, \end{aligned}$$

which are specified as linear combinations of SMEFT operators, the contributions of which are supplied in the simu_fac files.

Once a run card is prepared, the user simply follows the standard NNPDF4.0 pipeline to perform a fit. In particular, they should run vp-setupfit, n3fit, evolven3fit and postfit in sequence in order to obtain results. Analysis of the results can be achieved with a range of tools supplied with the release; further details are given below and on the website: https://hep-pbsp.github.io/SIMUnet/.

*The fixed PDF feature of* SIMUnet. The SIMUnet release also provides functionality for fits of the physical parameters ${\textbf {c}}$ alone, with the PDFs kept fixed (similar to tools such as SMEFiT or fitmaker in the case of the SMEFT Wilson coefficients). This is achieved simply by loading the weights of a previous neural network PDF fit,^5^ then keeping these weights fixed as the remaining parameters are fitted.

To specify this at the level of a SIMUnet run card, use the syntax:
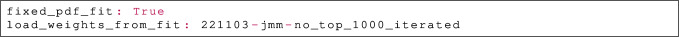


for example. Setting fixed_pdf_fit to True instructs SIMUnet to perform the fit keeping the weights of the PDF part of the network constant, and the namespace load_weights_from_fit tells SIMUnet which previous PDF fit to obtain the frozen weights from. The pipeline for a fit then proceeds exactly as in the case of a normal SIMUnet fit, beginning by running vp-setupfit and then n3fit. *However*, the user need not use evolven3fit at the subsequent stage, since the PDF grid is already stored and does not need to be recomputed; it does still need to be copied into the correct fit result directory though, which should be achieved using the vp-fakeevolve script via:
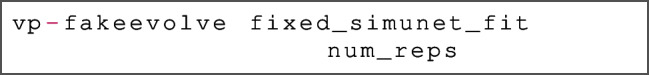


where fixed_simunet_fit is the name of the simultaneous fit that the user has just run, and num_reps is the number of replicas in the fit. The user *must* still run postfit after running the vp-fakeevolve script.

The ability to load weights from a previous fit can also be used to aid a simultaneous fit, by starting training from a good PDF solution already. For example, if we use the syntax:
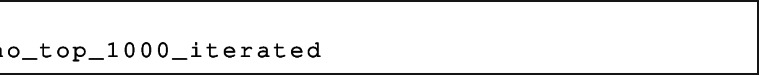


in a SIMUnet run card, then the weights of the PDF part of the neural network will be initialised to the weights of the appropriate replica from the previous PDF fit 221103-jmm-no_top_1000_iterated. *However*, since the namespace fixed_pdf_fit is set to False here, the fit will *still* be simultaneous, fitting both PDFs and physical parameters. Assuming that the resulting simultaneously-determined PDF fit is reasonably close to the previous PDF fit, this can significantly decrease training time.

SIMUnet *analysis tools.* The SIMUnet code is released with a full suite of analysis tools, which build on the tools already available in the NNPDF public code. These tools rely on the validphys analysis framework implemented in reportengine, and address exclusively the PDF aspect of the fits. They allow the user, for example, to generate PDF plots, luminosities, compare fits, and evaluate fit quality metrics, among many other things.

The SIMUnet code adds an extensive set of tools to study results in the SMEFT and assess the PDF-SMEFT interplay. These analysis tools are exclusively allocated in the simunet_analysis.py file, where the user can find documented functions to perform different tasks. In the context of EFT coefficients, SIMUnet calculates, among other things, their posterior distributions, Wilson coefficient bounds, correlations and pulls from the SM. We refer the reader to the new results presented in Sect. 4 to visualise the types of analysis that SIMUnet can perform.

## Closure tests and ‘contaminated’ fits within SIMUnet

The NNPDF4.0 *closure test* methodology, first described in Ref. [64] and described in much more detail in Ref. [65], is regularly deployed by the NNPDF collaboration to ensure that their methodology is working reliably. The SIMUnet code extends the capacity of the NNPDF closure test to run *contaminated* fits (as performed by the private code used for Ref. [36]) and closure tests probing the fit quality of *both* PDFs and physical parameters.

In Sect. 3.1 we begin with a brief review of the NNPDF4.0 methodology and identify the key assumption that SIMUnet relaxes, namely that the values of the physical parameters used to produce the artificial data are the same as those used in the subsequent PDF fit of the artificial data. We then proceed to describe in Sect. 3.2 how to produce the so-called *contaminated* fits, i.e. fits in which a New Physics model is injected in the artificial data, while the fit is done assuming the Standard Model. With this functionality, any users can test the robustness of any New Physics scenario against being fitted away in a PDF fit. We give usage details for running such fits using the SIMUnet code. Finally, in Sect. 3.3 we discuss how SIMUnet can perform closure tests for both PDFs and physical parameters, giving surety of the reliability of the SIMUnet methodology.

### Review of the NNPDF closure test methodology

The NNPDF closure test methodology begins by supposing that we are given Nature’s true PDFs at the initial scale ${\textbf {f}}_{\text {true}}(x,Q_0^2) = {\textbf {NN}}(x, \pmb {\omega }_{\text {true}})$,^6^ and true physical parameters ${\textbf {c}}_{\text {true}}$. Under this assumption, experimental data ${\textbf {D}}$ should appear to be a sample from the multivariate normal distribution:

3.1 $$\begin{aligned} {\textbf {D}} \sim {\mathcal {N}}({\textbf {T}}(\pmb {\omega }_{\text {true}}, {\textbf {c}}_{\text {true}}), \Sigma _{\text {exp}}), \end{aligned}$$

where $\Sigma _{\text {exp}}$ is the experimental covariance matrix, and ${\textbf {T}}$ is the theory prediction from the full discrete convolution formula Eq. (2.2) (*not* the SIMUnet prediction Eq. (2.6)). A sample from this distribution is called a set of *level 1 pseudodata* in the NNPDF closure test language. Given a set of level 1 pseudodata, we can attempt to recover the theory of Nature using the NNPDF4.0 methodology. Instead of fitting PDFs to experimental data, we can perform the fit replacing the experimental data with the level 1 pseudodata. If the resulting PDF fit has a spread covering the true PDF law ${\textbf {f}}_{\text {true}}$, we say that the methodology has passed this ‘closure test’.^7^

Importantly, closure tests are typically performed *only* fitting the true PDF law; in particular, the closure test usually assumes that the true physical parameters ${\textbf {c}}_{\text {true}}$ are known exactly and perfectly, that is, theory predictions for the fit take the form ${\textbf {T}}(\pmb {\omega }, {\textbf {c}}_{\text {true}})$ where the PDF parameters $\pmb {\omega }$ are variable and fitted to the pseudodata, but the physical parameters ${\textbf {c}}_{\text {true}}$ are fixed. With SIMUnet, more options become available, as described below.

### BSM-contaminated fits with SIMUnet

In Ref. [36], closure tests were performed using the standard closure test methodology, but introducing a mismatch between the theory used as the ‘true’ underlying law of Nature and the theory used in the fit; that is, theory predictions for the closure test fit took the form ${\textbf {T}}(\pmb {\omega }, {\textbf {c}}^*)$ where ${\textbf {c}}^* \ne {\textbf {c}}_{\text {true}}$. The authors of Ref. [36] call the result of such a fit a *contaminated* fit; in that work, this is applied particularly to the case that the fake data is generated with New Physics beyond the Standard Model, but the PDFs are fitted assuming the Standard Model, i.e. ${\textbf {c}}_{\text {true}}$ is a vector of BSM parameters, and ${\textbf {c}}^*$ is the corresponding vector where these BSM parameters are set to the values they would take in the Standard Model. In the case that the contaminated fit quality is not noticeably poor, the PDFs are said to *reabsorb* New Physics, and it is shown in Ref. [36] that this can lead to dangerous consequences in subsequent searches for New Physics.

The SIMUnet code provides the capacity to easily perform contaminated fits, assuming that the underlying theory for the level 1 pseudodata is generated according to the linear *K*-factor form given by Eq. (2.6), i.e. the level-1 pseudodata is generated as a sample from the multivariate normal distribution with mean:

3.2 $$\begin{aligned} {\textbf {T}}(\pmb {\omega }_{\text {true}}, {\textbf {c}}^*) \odot \left[ {\textbf {1}} + {\textbf {K}}_{\text {fac}}(\pmb {\omega }^*) ({\textbf {c}}_{\text {true}} - {\textbf {c}}^*) \right] , \end{aligned}$$

where $\pmb {\omega }^*, {\textbf {c}}^*$ are the reference values of the PDF parameters and physical parameters assumed in the relevant model of the relevant simu_fac files. This is effected in the SIMUnet code simply by interceding in the standard NNPDF4.0 generation of the level 1 pseudodata, by replacing the central values to which Gaussian noise are added by those presented in Eq. (3.2).

Note that the code can be easily modified so that any New Physics can be injected in the level 1 pseudodata, without relying on the SMEFT expansion, but simply by computing the multiplicative factor associated to the modification of the theoretical prediction for the observable included in the fit due to the presence of a given New Physics mode. Hence, the procedure is completely general, in principle, and does not rely on any EFT expansion.

*Code usage.* In terms of usage, a contaminated fit using the SIMUnet code should be based on an NNPDF4.0 closure test run card; in particular, the closuretest namespace must be specified. The required SIMUnet additions to such a run card are twofold and described as follows. First, the dataset_inputs must be modified with a new key, for example:
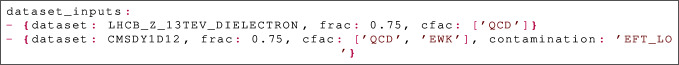


Here, two datasets are included: (i) the LHCB_Z_13TEV_DIELECTRON dataset has no additional keys compared to a standard NNPDF4.0 closure test run card, and hence the level 1 pseudodata for this set is generated normally; (ii) the CMSDY1D12 dataset has an additional key, contamination, set to the value ’EFT_LO’, which tells SIMUnet to base the level 1 pseudodata for this set on the predictions given by Eq. (3.2), using parameters drawn from the model EFT_LO in the relevant simu_fac file. The precise values of the true physical parameters used in Eq. (3.2) are specified as part of the closuretest namespace, which is the second modification required to a standard closure test run card: 
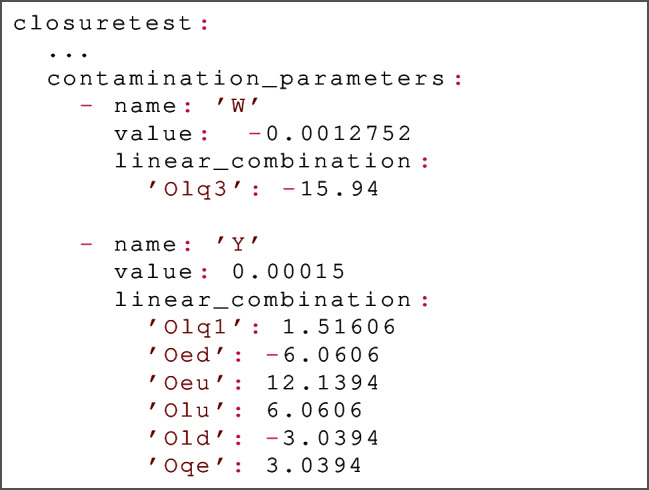


In the example above we have specified that we inject a New Physics model that belongs to the so-called *universal theories* in which the parameters ${\hat{W}}$ and ${\hat{Y}}$, which are a combination of four-fermion operators defined for example in Refs. [33, 35, 36], are set the values given in this example. The precise definitions of the linear combinations in this example are given by:

$$\begin{aligned} {\hat{W}}\,{\mathcal {O}}_W= & {} (-0.0012752) \times \left( -15.94\, {\mathcal {O}}_{lq3}\right) ,\\ {\hat{Y}}\,{\mathcal {O}}_Y= & {} (0.00015) \times \left( 1.51606\, {\mathcal {O}}_{lq1} \right. \nonumber \\ {}{} & {} -6.0606\,{\mathcal {O}}_{ed} +12.1394\,{\mathcal {O}}_{eu}\\{} & {} +6.0606\,{\mathcal {O}}_{lu}-3.0394\,{\mathcal {O}}_{ld} \\{} & {} \left. +3.0394\,{\mathcal {O}}_{qe}\right) . \end{aligned}$$

**Fig. 2:**
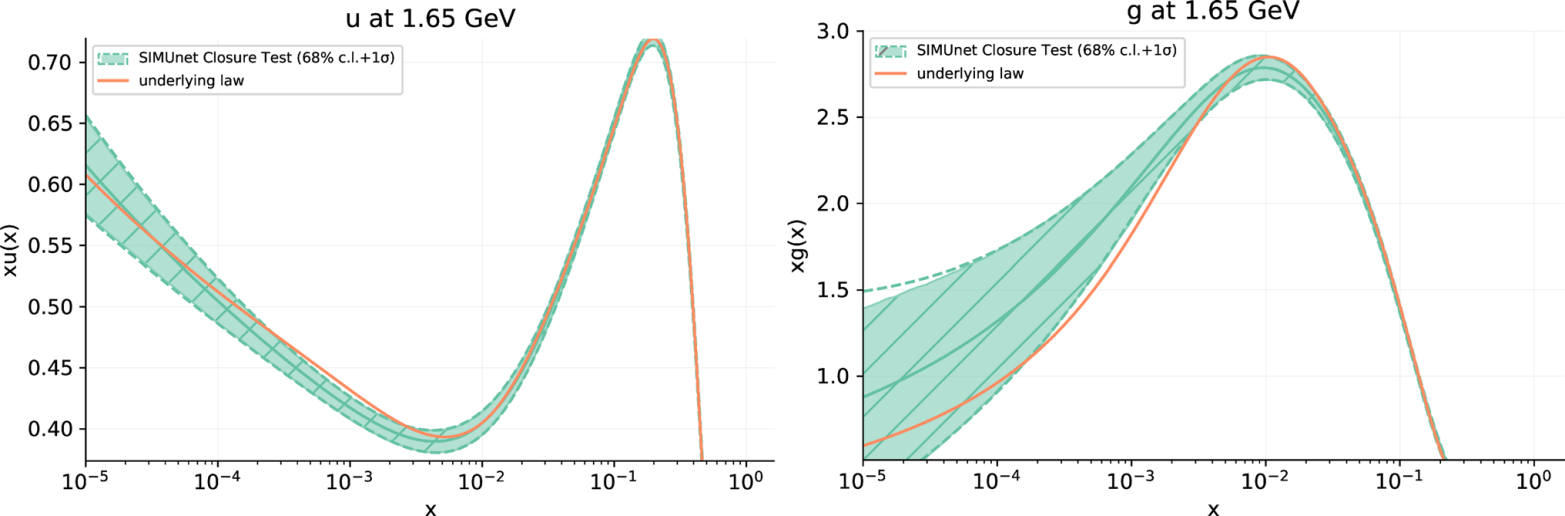
The up quark (left) and gluon (right) PDFs obtained from the closure test framework in which both the underlying PDF set and Wilson coefficients are known. Shown in orange is the PDF replica used as the underlying law which generates the fake data used to train our model. The resulting PDFs are shown in green along with their 68% confidence level bands. The fake data generated by the underlying law is subsequently modified so as to encode the ($c_{tG}$, $c_{lq}^{(3)}$) = (1, 0.08) condition

### Closure tests with SIMUnet

The ‘contamination’ feature of the SIMUnet code allows the user not only to study whether a theory bias can be reabsorbed into the PDFs, but can be used in conjunction with the simultaneous fit feature to perform closure tests on the simultaneous fits of PDFs and SMEFT Wilson coefficients. The closure test validates how much SIMUnet is able to replicate, not only fixed Wilson coefficients, but also a known underlying PDF. In the context of a simultaneous fitting methodology a ‘level 2’ closure test amounts to generating SM predictions using a known PDF set (henceforth referred to as the underlying law) and mapping these to SMEFT observables by multiplying the SM theory predictions with SMEFT *K*-factors scaled by a previously determined choice of Wilson coefficients. These SMEFT observables, generated by the underlying law, replace the usual MC pseudodata replicas, and are used to train the neural network in the usual way.

Through this approach we are not only able to assess the degree to which the parametrisation is able to capture an underlying choice of Wilson coefficients, but also ensure it is sufficiently flexible to adequately replicate a known PDF.

*Closure test settings.* In the rest of the section, we present the results of a simultaneous closure test the validates the SIMUnet methodology in its ability to produce an underlying law comprising both PDFs and Wilson coefficients. In this example we set:

$$\begin{aligned} \omega _{\textrm{true}}&= \texttt {NNPDF40\_nnlo\_as\_0118\_1000},\\ {\textbf {c}}_{\textrm{true}}&= \begin{pmatrix} c_{tG} \\ c_{lq}^{(3)} \end{pmatrix} = \begin{pmatrix} 1 \\ 0.08 \end{pmatrix}. \end{aligned}$$

That is, NNPDF4.0 serves as the true underlying PDF law, and all Wilson coefficients are set to zero except $c_{tG}$ and $c_{lq}^{(3)}$. Note that the true values of the Wilson coefficients that we choose in this test are outside the 95% C.L. bounds on the Wilson coefficients that were found in previous analyses [21, 33] corresponding to $c_{tG}\in (-0.18,0.17)$ and $c_{lq}^{(3)}\in (-0.07,0.02)$ respectively, hence the success of the closure test is non-trivial.

The results of the closure test for the gluon and up quark PDFs at the parametrisation scale are presented in Fig. 2. Here we can see that the combination layer is capturing the data’s dependence on the Wilson coefficients, whilst the complementary PDF sector of the network architecture captures the data’s dependence on the underlying PDF. The combination layer, in effect, subtracts off the EFT dependence, leaving behind the pure SM contribution for the PDF sector to parameterise.

**Fig. 3:**
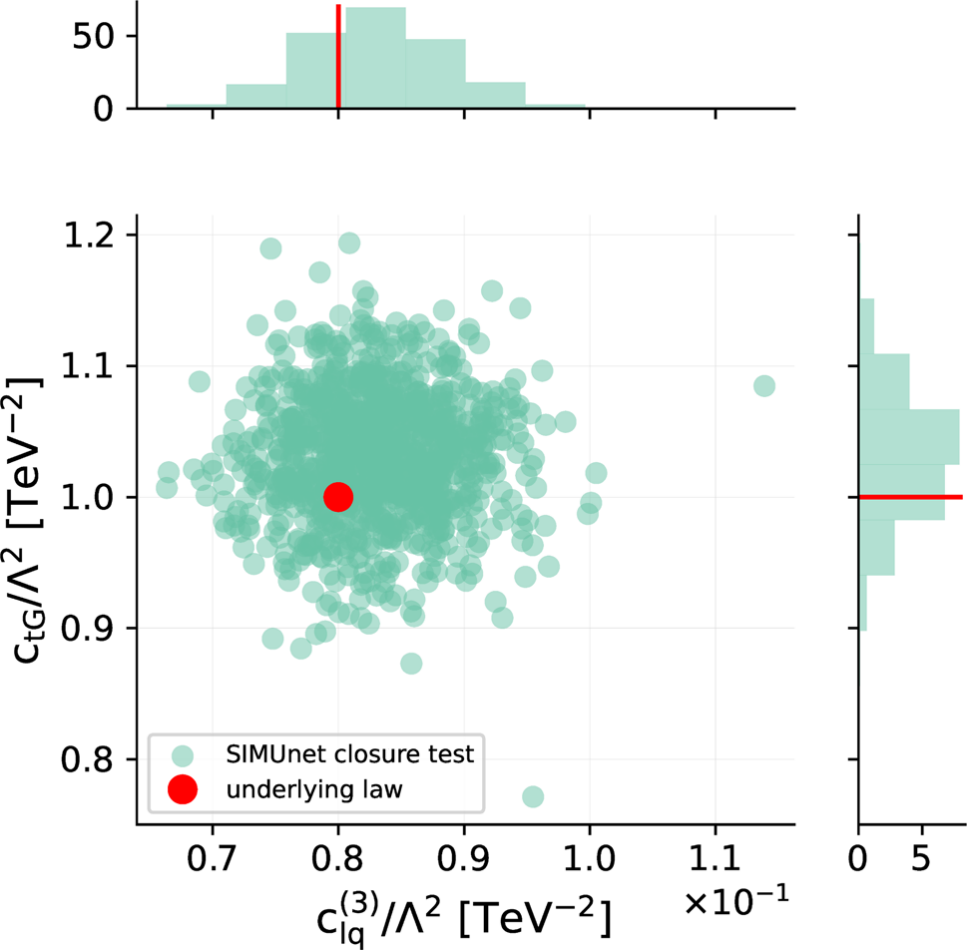
Result of the closure testing framework for our methodology. The distribution of ($c_{tG}$,$c_{lq}^{(3)}$) when fitting to data that has been modified by setting ($c_{tG}$,$c_{lq}^{(3)}$) = (1, 0.08). The upper and right panels show the histograms for the distribution of the best fit values in their respective directions

The corresponding results for the Wilson coefficients $c_{tG}$ and $c_{lq}^{(3)}$ are displayed in Fig. 3. Both figures demonstrate that the parametrisation successfully captures the underlying law for both the PDFs and Wilson coefficients. To verify the robustness of these findings and rule out random fluctuations, we conducted the closure test 25 times, each time using different level 1 pseudodata; the results consistently remained stable and aligned with those presented above.

## A first global simultaneous fit of SMEFT WCs and PDFs

In this section we present the first global simultaneous fit of SMEFT WCs and PDFs, including Deep-Inelastic-Scattering, Drell–Yan, top, jets, Higgs, diboson and EW precision observables. This analysis can be now performed thanks to the SIMUnet methodology that we have described so far. We start in Sect. 4.1 by listing the data that have been included, and giving details of their implementation. In Sect. 4.2 we present the result of the fixed-PDF analysis, producing a global SMEFT fit of a large number of datapoints from the Higgs, top, and EW sectors ($n_{\textrm{data}}\,=\,366$) to a set of $n_{\textrm{op}}\,=\,40$ SMEFT operators. Finally in Sect. 4.3 we present the simultaneous PDF and SMEFT global fit ($n_{\textrm{data}}\,=\,4985$). We compare the resulting PDFs and SMEFT bounds to the SM PDFs and to the SMEFT bounds obtained in the fixed-PDF fit respectively, hence assessing the effect of the interplay between PDFs and SMEFT effects on both our description of the proton and on New Physics bounds.

### Experimental data

The dataset explored in this study builds on those already implemented in the high-mass Drell–Yan analysis presented in Refs. [33, 35] and on the top quark analysis presented in Ref. [21]. These studies extended the datasets included in the global NNPDF4.0 [40] analysis, by adding observables that enhance the sensitivity to the SMEFT and that constrain PDFs in the Drell–Yan and in the top sector respectively.

**Table 1:** Measurements of electroweak precision observables included in SIMUnet. The columns contain information on the centre-of-mass energy, the observable, the integrated luminosity, the number of data points, the dataset name as implemented in SIMUnet and the source

Exp.	$\mathbf {\sqrt{s}}$ (TeV)	Observable	${\mathcal {L}}$ (${\textrm{fb}}^{-1}$)	${\textbf{n}_{\mathbf {{dat}}}}$	Dataset name	References
LEP	0.250	Z observables		19	LEP_ZDATA	[66]
LEP	0.196	${\mathcal {B}}(W \rightarrow l^{-} {\bar{v}}_l)$	3	3	LEP_BRW	[67]
LEP	0.189	$\sigma (e^+ e^- \rightarrow e^+ e^-)$	3	21	LEP_BHABHA	[67]
LEP	0.209	$\hat{\alpha }^{(5)}(M_Z)$	3	1	LEP_ALPHAEW	[68]

The NNPDF4.0 NNLO analysis [40] included $n_\textrm{data}\,=\,4618$ data points corresponding to a wide variety of processes in deep-inelastic lepton-proton scattering (from the HERA *ep* collider and from fixed-target experiments such as NMC, SLAC, BCDMS, CHORUS and NuTeV), fixed-target DY data from the Fermilab E605 and E866 experiments, hadronic proton–antiproton collisions from the Tevatron, and proton–proton collisions from LHC. The LHC data in turn include inclusive gauge boson production data; *Z*- and *W*-boson transverse momentum production data, single-inclusive jet and di-jets production data, as well as gauge boson with jets and inclusive isolated photon production data and top data. In Refs. [33, 35] the high-mass Drell–Yan sector of the NNPDF4.0 analysis was augmented by two extra measurements taken by CMS at $\sqrt{s}=8,13$ TeV, for a total of 65 extra datapoints. In Ref. [21], besides inclusive and differential $t{\bar{t}}$ cross sections and *t*-channel single top production already implemented in NNPDF4.0, more observables were included, such as $t{\bar{t}}$ production asymmetries, *W*-helicity fractions, associated top pair production with vector bosons and heavy quarks, including ${\bar{t}}t Z$, ${\bar{t}}t W$, ${\bar{t}}t \gamma$, ${\bar{t}}t{\bar{t}}t$, ${\bar{t}}t{\bar{b}}b$, *s*-channel single top production, and associated single top and vector boson production, bringing the total number of datapoints to $n_{\textrm{data}}\,=\,4710$.

In this present study, we further extend the datasets by probing electroweak precision observables (EWPOs), the Higgs sector and the diboson sector. The datasets are described below and details such as the centre-of-mass energy, the observable, the integrated luminosity, the number of data points, the dataset name as implemented in SIMUnet and the source are given in Tables 1, 2 and 3 respectively.

We remind the reader that, thanks to the functionality of SIMUnet that enables users to include PDF-independent observables as well as to freeze PDFs for the observables in which the PDF dependence is mild, we can add observables such as the measurement of $\alpha _e$ that are completely independent of PDFs (but which are affected by SMEFT corrections) or Higgs signal strengths and Simplified Template Cross Section (STXS) measurements that are mildly dependent on PDFs but are strongly dependent on the relevant SMEFT-induced corrections.

To summarise, in this analysis we include a total of **4985 datapoints**, of which 366 are affected by SMEFT corrections. Among those 366 datapoints, 210 datapoints are PDF independent. Hence our pure PDF analysis performed with SIMUnet includes $(4985-210) = 4775$ datapoints and is equivalent to the NNPDF4.0 global analysis augmented by 78 datapoints from the top sector [21] and by 65 datapoints measuring the high-mass Drell–Yan tails [33, 35]. Our pure SMEFT-only analysis performed with SIMUnet includes 366 datapoints, while our simultaneous PDF-SMEFT fit includes 4985 datapoints.

**EWPOs:** In Table 1, we list EWPOs included in this study. The dataset includes the pseudo-observables measured on the *Z* resonance by LEP [66], which include cross sections, forward-backward and polarised asymmetries. Branching ratios of the decay of the *W* boson into leptons [67] are also included, along with the LEP measurement of the Bhabha scattering cross section [67]. Finally we include the measurement of the effective electromagnetic coupling constant [68], for a total of **44 datapoints**. These datasets and their SMEFT predictions are taken from the public SMEFiT code [27, 69]. These datapoints are all PDF independent, hence they directly affect only the SMEFT part of the fits.

**Higgs sector:** In Table 2, we list the Higgs sector datasets included in this study. The Higgs dataset at the LHC includes the combination of Higgs signal strengths by ATLAS and CMS for Run 1, and for Run 2 both signal strengths and STXS measurements are used. ATLAS in particular provides the combination of stage 1.0 STXS bins for the 4*l*, $\gamma \gamma$, *WW*, $\tau \tau$ and $b{\bar{b}}$ decay channels, while for CMS we use the combination of signal strengths of the 4*l*, $\gamma \gamma$, *WW*, $\tau ^-\tau ^+$, $b{\bar{b}}$ and $\mu ^-\mu ^+$ decay channels. We also include the $H \rightarrow Z\gamma$ and $H \rightarrow \mu ^-\mu ^+$ signal strengths from ATLAS, for a total of **73 datapoints**. The Run I and CMS datasets and their corresponding predictions are taken from the SMEFiT code [27, 69], whereas the STXS observables and signal strength measurements of $H \rightarrow Z\gamma$ and $H \rightarrow \mu ^-\mu ^+$ are taken from the fitmaker[25] code. The signal strength’s dependence on the PDFs is almost completely negligible, given that the PDF dependence cancels in the ratio, hence all the datapoints are treated as PDF independent and are directly affected only by the SMEFT Wilson coefficients.

**Table 2:** Same as 1 for the measurements of the Higgs sector

Exp.	$\mathbf {\sqrt{s}}$ (TeV)	Observable	${\mathcal {L}}$ (${\textrm{fb}}^{-1}$)	${\textbf{n}_{\mathbf {{dat}}}}$	Dataset name	References
ATLAS and CMS	7 and 8	$\mu _{H \rightarrow \mu ^+ \mu ^-}$	5 and 20	22	ATLAS_CMS_SSinc_RunI	[70]
CMS	13	$\mu _{H}$	35.9	24	CMS_SSINC_RUNII	[71]
ATLAS	13	$\mu _{H}$	80	25	ATLAS_STXS_RUNII	[72]
ATLAS	13	$\mu _{H \rightarrow Z \gamma }$	139	1	ATLAS_SSINC_RUNII_ZGAM	[73]
ATLAS	13	$\mu _{H \rightarrow \mu ^+ \mu ^-}$	139	1	ATLAS_SSINC_RUNII_MUMU	[74]

**Diboson sector:** In Table 3, we list the diboson sector datasets included in this study. We have implemented four measurements from LEP at low energy, three from ATLAS and one from CMS both at 13 TeV. The LEP measurements are of the differential cross-section of $e^+ e^- \rightarrow W^+ W^-$ as a function of the cosine of the *W*-boson polar angle. The ATLAS measurements are of the differential cross-section of $W^+ W^-$ as a function of the invariant mass of the electron-muon pair and of the transverse mass of the *W*-boson. The third ATLAS measurement is of the differential cross-section of *Zjj* as a function of the azimuthal angle between the two jets, $\Delta \phi _{jj}$. Although this is not a diboson observable, it is grouped here as this observable constrains operators typically constrained by the diboson sector, such as the triple gauge interaction $O_{WWW}$. The CMS dataset is a measurement of the differential cross-section of *WZ* as a function of the transverse momentum of the *Z*-boson, for a total of **82 datapoints**. Almost all datasets and corresponding predictions in this sector are taken from the SMEFiT code [27, 69], except for the ATLAS measurement of *Zjj* production, taken from the fitmaker[25] code. While the LEP measurements are PDF-independent by definition, the LHC measurements of diboson cross sections do depend on PDFs. However, their impact on the PDFs is extremely mild, due to much more competitive constraints in the same Bjorken-*x* region given by other measurements such as single boson production and single boson production in association with jets. Therefore we consider all diboson measurements to be PDF-independent, i.e. the PDFs are fixed and their parameters are not varied in the computation of these observables.

**Table 3:** Same as 1 for the measurements of the diboson sector

Exp.	$\mathbf {\sqrt{s}}$ (TeV)	Observable	${\mathcal {L}}$ (${\textrm{fb}}^{-1}$)	${\textbf{n}_{\mathbf {{dat}}}}$	Dataset name	References
LEP	0.182	$d \sigma _{WW} / d cos(\theta _W)$	0.164	10	LEP_EEWW_182GEV	[67]
LEP	0.189	$d \sigma _{WW} / d cos(\theta _W)$	0.588	10	LEP_EEWW_189GEV	[67]
LEP	0.198	$d \sigma _{WW} / d cos(\theta _W)$	0.605	10	LEP_EEWW_198GEV	[67]
LEP	0.206	$d \sigma _{WW} / d cos(\theta _W)$	0.631	10	LEP_EEWW_206GEV	[67]
ATLAS	13	$d \sigma _{W^+W^-}/d m_{e \mu }$	36.1	13	ATLAS_WW_13TeV_2016_MEMU	[75]
ATLAS	13	$d \sigma _{WZ} / d m_{T}$	36.1	6	ATLAS_WZ_13TeV_2016_MTWZ	[76]
ATLAS	13	$d \sigma (Zjj)/d \Delta \phi _{jj}$	139	12	ATLAS_Zjj_13TeV_2016	[77]
CMS	13	$d \sigma _{WZ} / d p_{T}$	35.9	11	CMS_WZ_13TeV_2016_PTZ	[77]

**SMEFT Wilson coefficients:**

We include a total of 40 operators from the dimension-6 SMEFT in our global analysis of the measurements of the Higgs, top, diboson and electroweak precision observables outlined above.

**Fig. 4:**
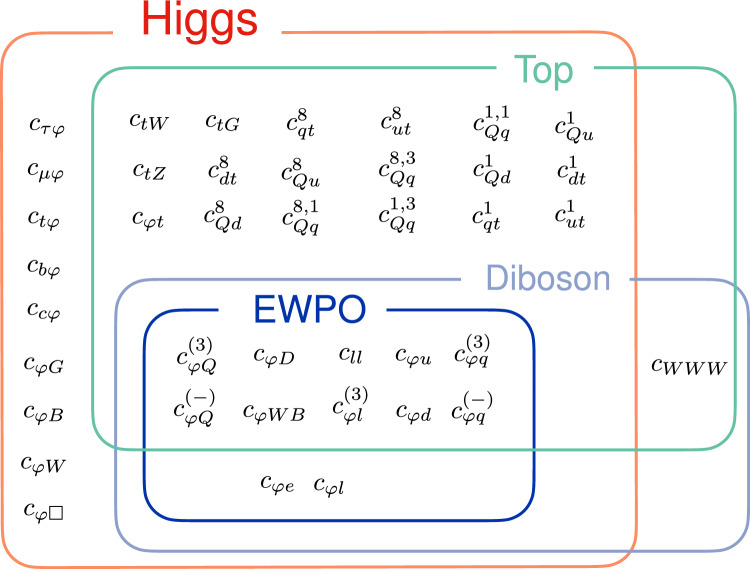
Schematic representation of the data categories included in this analysis and of their overlapping dependences on the 40 dim-6 SMEFT operators included in our global analysis, adapted from Ref. [25]

In Fig. 4 we display the SMEFT Wilson coefficients included in our analysis, following the operator conventions of Refs. [21, 26]. The schematic diagram, adapted from Ref. [25], demonstrates the sectors of data affected by each WC and highlights the interconnected nature of the global SMEFT determination. The overlaps between the different rectangles show explicitly how a given operator contributes to several data categories. For example, $O_{WWW}$, contributes to both the diboson sector and the top sector (through its contribution to *tZq* production), while the operators $O_{\varphi e}$,$O_{\varphi l}$ contribute to the Higgs, diboson and electroweak sectors, but have no effect on the top sector.

### Global SMEFT fit

We present the results of this new global fit in two parts, beginning with a discussion of the result of the fixed PDF fit, that is a global fit purely of the SMEFT Wilson coefficients. In Sect. 4.3 we will present the results of the simultaneous global fit of SMEFT WCs and will compare the results to those obtained here.

The global analysis is performed at linear order in the SMEFT operator coefficients, accounting for the interference between the SM and the insertion of a dimension-6 SMEFT operator. The linear constraints can be viewed as provisional for operators where quadratic contributions are non-negligible. Nevertheless, keeping those operators in the global fit typically yields conservative marginalised limits that allows one to assess the impact on other operators and whether this is significant to a first approximation. An analysis including quadratic contributions requires a new methodology that does not rely on the Monte-Carlo sampling method to propagate uncertainties [78], given that the latter fails at reproducing the Bayesian confidence intervals once quadratic corrections are dominant, as described in App. E of Ref. [21].

**Fig. 5:**
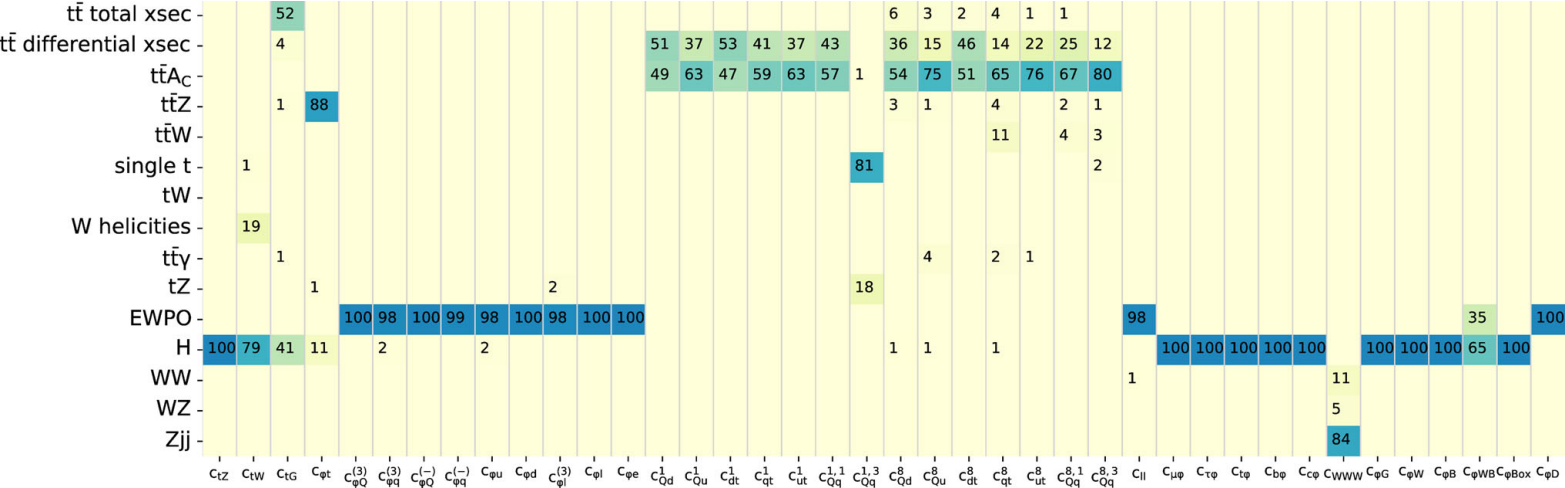
Relative constraining power (in %) on each of the operators for each of the processes entering the fit, as defined in Eq. (4.2)

**Fig. 6:**
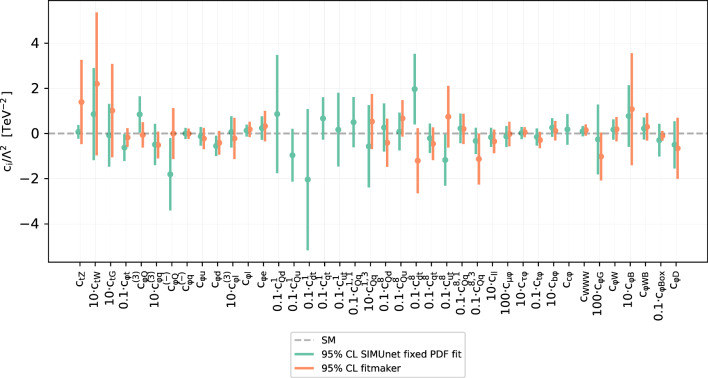
Comparison of the bounds for the SMEFT Wilson coefficients determined in the pure SMEFT analysis presented in this work and the fitmaker analysis [25]. The dashed horizontal line is the SM prediction. In each bound, the dot represents the best-fit value and the 95% CL interval is constructed from the envelope of two standard deviations away from this best-fit value

Our analysis comprises $n_{\textrm{op}} = 40$ operators in a simultaneous combination of the constraints from $n_\textrm{data}\,=\,366$ datapoints from the Higgs, EWPO, diboson and top sectors. Note that in the SIMUnet code there are more SMEFT operators implemented, in particular the set of four-fermion operators that affect Drell–Yan and DIS data. As demonstrated in [32, 33], the effect of the four fermion operators in DIS and on the current Drell–Yan data, including the high-mass Drell–Yan data included in NNPDF4.0 is not significant enough for this data to provide strong constraints on the relevant set of four-fermion operators. On the contrary, the HL-LHC projections, which include both neutral and charged current Drell–Yan data have a strong potential in constraining such operators [35]. As a results, Drell–Yan data are not affected by SMEFT corrections in this current analysis, but we leave the user the opportunity to include such effects straightforwardly, should the measurements by the LHC increase the constraining potential, or should the user combine those with flavour observables, as is done in Ref. [31].

In the following, we also omit from the results the 4 heavy quark production datasets (four tops and $t{\bar{t}}b{\bar{b}}$). Their effect is practically negligible on all the operators affecting the other sectors and they would simply constrain 2 directions in the 5-dimensional space of the four heavy fermion operators. These observables are therefore *de facto* decoupled from the rest of the dataset.

The input PDF set, which is kept fixed during the pure SMEFT fit, is the nnpdf40nnlo_notop baseline, corresponding to the NNPDF4.0 fit without the top data, to avoid possible contamination between PDF and EFT effects in the top sector [21].

**Fig. 7:**
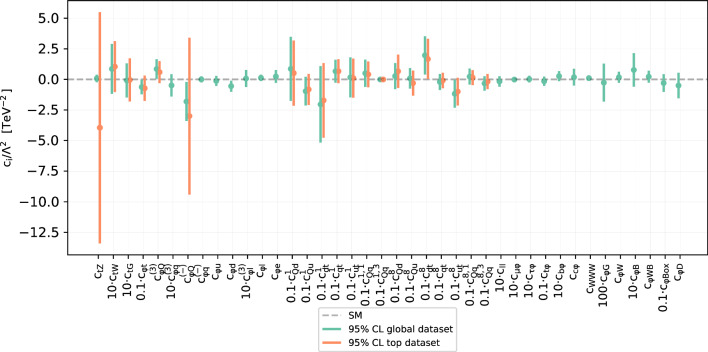
Same as Fig. 6, now comparing the 95% CL bounds obtained in the SMEFT top analysis of Ref. [21] (old dataset) with the 95% CL bounds obtained in this global SMEFT analysis

The sensitivity to the EFT operators of the various processes entering the fit can be evaluated by means of the Fisher information, which, for *N* EFT coefficients, is an $N \times N$ matrix given by

4.1 $$\begin{aligned} F_{ij} = {L_{i}}^T (\Sigma _{\text {exp}})^{-1} L_{j}. \end{aligned}$$

Here $L_{i}$ is the vector of the linear contributions to the observables of the *i*-th SMEFT Wilson coefficient, entering into the theory prediction *T*(*c*) as $T(c)_{\alpha } = T(c)^\textrm{SM}_{\alpha } + (L_i)_{\alpha } c_i$, where the index $\alpha$ runs over the datapoints in the fit, and $\Sigma _{\text {exp}}$ is the experimental covariance matrix. The covariance matrix in the space of SMEFT coefficients $C_{ij}$, by the Cramér-Rao bound, satisfies $C_{ij} \ge (F^{-1})_{ij}$, hence the larger the entries of the Fisher information matrix, the smaller the possible uncertainties of the SMEFT coefficients and, therefore, the bigger the constraining power of the relevant dataset.

**Table 4:** The 95% confidence intervals on three key Wilson coefficients included in this analysis

Operator	Global fit	Top fit
$c_{\phi t}$	($-$ 13, $-$ 0.22)	($-$ 18, 3.1)
$c_{tZ}$	($-$ 0.18, 0.37)	($-$ 13, 5.5)
$c_{\phi Q}^{(-)}$	($-$ 3.6, $-$ 0.042)	($-$ 9.4, 3.4)

In Fig. 5 we use the Fisher information to assess the relative impact of each sector of data included in our analysis on the SMEFT parameter space by plotting the relative percentage constraining power of the dataset *D* via:

4.2 $$\begin{aligned}{} & {} \text {relative constraining power of }D\text { on operator }c_i\nonumber \\{} & {} \quad = F_{ii}(D) \bigg / \displaystyle \sum _{\text {sectors }D'} F_{ii}(D'), \end{aligned}$$

which gives a general qualitative picture of some of the expected behaviour in the global fit. In Fig. 5 we observe that the coefficients $c_{tZ}$, $c_{tW}$ are dominantly constrained by the Higgs sector, while the coefficient $c_{tG}$ is now constrained both by the $t {\bar{t}}$ total cross sections and by the Higgs measurements, as expected. Higgs measurements also provide the dominant constraints on the bosonic coefficients $c_{\varphi G}$, $c_{\varphi W}$, $c_{\varphi B}$, $c_{\varphi \Box }$ and on the Yukawa operators. Coefficients coupling the Higgs to fermions, $c_{\varphi Q}^{(3)}$, $c_{\varphi q}^{(3)}$, $c_{\varphi Q}^{(-)}$, $c_{\varphi q}^{(-)}$, $c_{\varphi u}$, $c_{\varphi d}$, $c_{\varphi l}^{(3)}$ and $c_{\varphi e}$, receive their dominant constraining power from the electroweak precision observables, as does the four-fermion coefficient $c_{ll}$. The coefficient $c_{WWW}$ is constrained by the diboson sector, with measurements of *Zjj* production providing the leading constraints at linear order in the SMEFT. From the top sector of the SMEFT, the four-fermion operators are constrained by measurements of top quark pair production total cross sections, differential distributions and charge asymmetries. The exception to this is $c_{Qq}^{(1,3)}$, which is constrained by both single top production and single top production in association with a *Z* boson. Overall we find qualitatively similar results to those shown in Refs. [25, 26].

To assess the strength of the bounds on the Wilson coefficients that we find in this work and for reference, in Fig. 6 we compare the bounds that we obtain here against the fixed-PDF SMEFT analysis presented by the fitmaker collaboration [25], which is the global public SMEFT analysis that currently includes all sectors that are considered here.

We observe that the bounds are comparable, with a few differences. First of all fitmaker uses LO QCD predictions for the SMEFT corrections in the top sector, hence most of the singlet four fermion operators in the top sector are not included, while SIMUnet uses NLO QCD predictions and obtains bounds on those, although weak [79]. Moreover, while the other bounds overlap and are of comparable size, the ${{{\mathcal {O}}}}_{tZ}$ is much more constrained in the SIMUnet analysis, as compared to the fitmaker one. This is due to the combination of all operators and observables, which collectively improves the constraints on this specific operator, thanks to the interplay between $O_{tZ}$ and the other operators that enter the same observables.

In Fig. 7 we show the comparison between the results of this current global SMEFT analysis against the SMEFT analysis of the top sector presented in [21].

In general, the results are very compatible, with bounds on most Wilson coefficients comparable between the two fits. However, the increased dataset size results in a marked improvement on the constraints of several Wilson coefficients, in particular $c_{\phi t}$, $c_{tZ}$ and $c_{\phi Q}^{(-)}$; see Table 4 for comparisons.

We observe that the bounds on $c_{\phi t}$ become more stringent by nearly a factor of 2, due to the extra constraints that come from the Higgs sector. Analogously, the Higgs constrains reduce the size of the bounds on $c_{\phi Q}^{(-)}$ by a factor of 3. The most remarkable effect is seen in $c_{tZ}$ which is now strongly constrained by the top loop contribution to the $H\rightarrow \gamma \gamma$ decay, for which we include experimental information on the signal strength from LHC Run II.

**Fig. 8:**
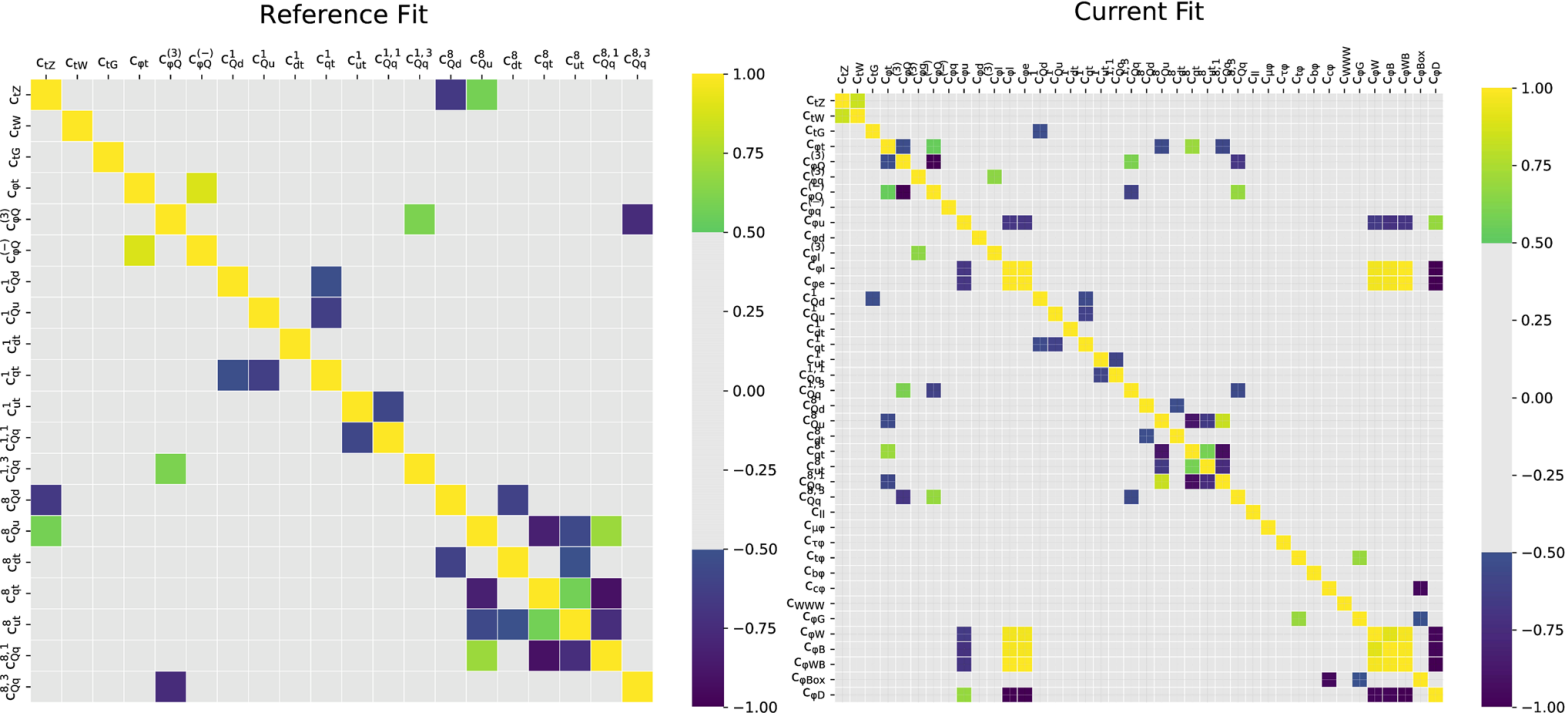
The values of the correlation coefficients evaluated between all pairs of Wilson coefficients entering the SMEFT fit of the top-only analysis (left) and the global analysis (right)

It is interesting to compare the correlations between Wilson coefficients evaluated in this analysis to the top-only SMEFT analysis of Ref. [21]. In Fig. 8 we note that the additional sectors, particularly the Higgs one, introduce a degree of anti-correlation between $c_{\phi Q}^{(3)}$ and $c_{\phi t}$ and between $c_{Q q}^{(8,3)}$ and $c_{Q q}^{(1,3)}$, while the one between $c_{u t}^{8}$ and $c_{d t}^{8}$ goes away in the global analysis. In the EW sector, we find very strong correlations, in agreement with similar studies in the literature. This suggests that there is room for improvement in the sensitivity to the operators affecting EW observables once more optimal and targeted measurements are employed in future searches [80, 81].

Finally we display the correlations observed between the PDFs and Wilson coefficients. The PDF-EFT correlation coefficient for a Wilson coefficient *c* and a PDF *f*(*x*, *Q*) at a given *x* and $Q^2$ is defined as

4.3 $$\begin{aligned}{} & {} \rho \left( c, f(x,Q^2)\right) \nonumber \\{} & {} \quad =\frac{\big \langle c^{(k)}f^{(k)}(x,Q^2)\big \rangle _k - \big \langle c^{(k)}\big \rangle _k \big \langle f^{(k)}(x,Q^2)\big \rangle _k }{\sqrt{\big \langle (c^{(k)})^2\big \rangle _k \!- \!\big \langle c^{(k)}\big \rangle _k^2}\sqrt{\big \langle \left( f^{(k)}(x,Q^2)\right) ^2\big \rangle _k \!-\! \big \langle f^{(k)}(x,Q^2)\big \rangle _k^2}} \,,\nonumber \\ \end{aligned}$$

where $c^{(k)}$ is the best-fit value of the Wilson coefficient for the *k*-th replica and $f^{(k)}$ is the *k*-th PDF replica computed at a given *x* and *Q*, and $\big \langle \cdot \big \rangle _k$ represents the average over all replicas. We compute the correlation between a SM PDF and the Wilson coefficients of this new global analysis. By doing so we have a handle on which PDF flavours and kinematical regions are strongly impacted by the presence of a given SMEFT-induced correction to the SM theoretical predictions, thus exhibiting a potential for interplay in a simultaneous EFT-PDF determination.

**Fig. 9:**
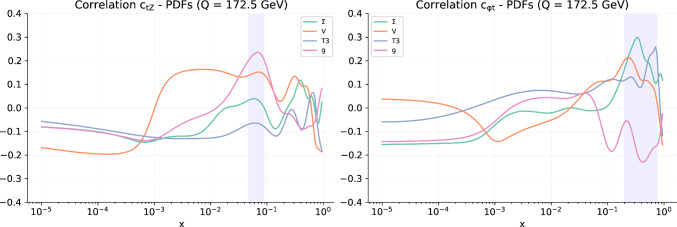
The value of the correlation coefficient $\rho$ between the PDFs and selected EFT coefficients as a function of *x* and $Q=172.5$ GeV. We show the results for the gluon, the total singlet $\Sigma$, total valence *V*, and non-singlet triplet $T_3$ PDFs. We provide results for representative EFT coefficients, namely $c_{tZ}$ and $c_{\phi t}$

**Fig. 10:**
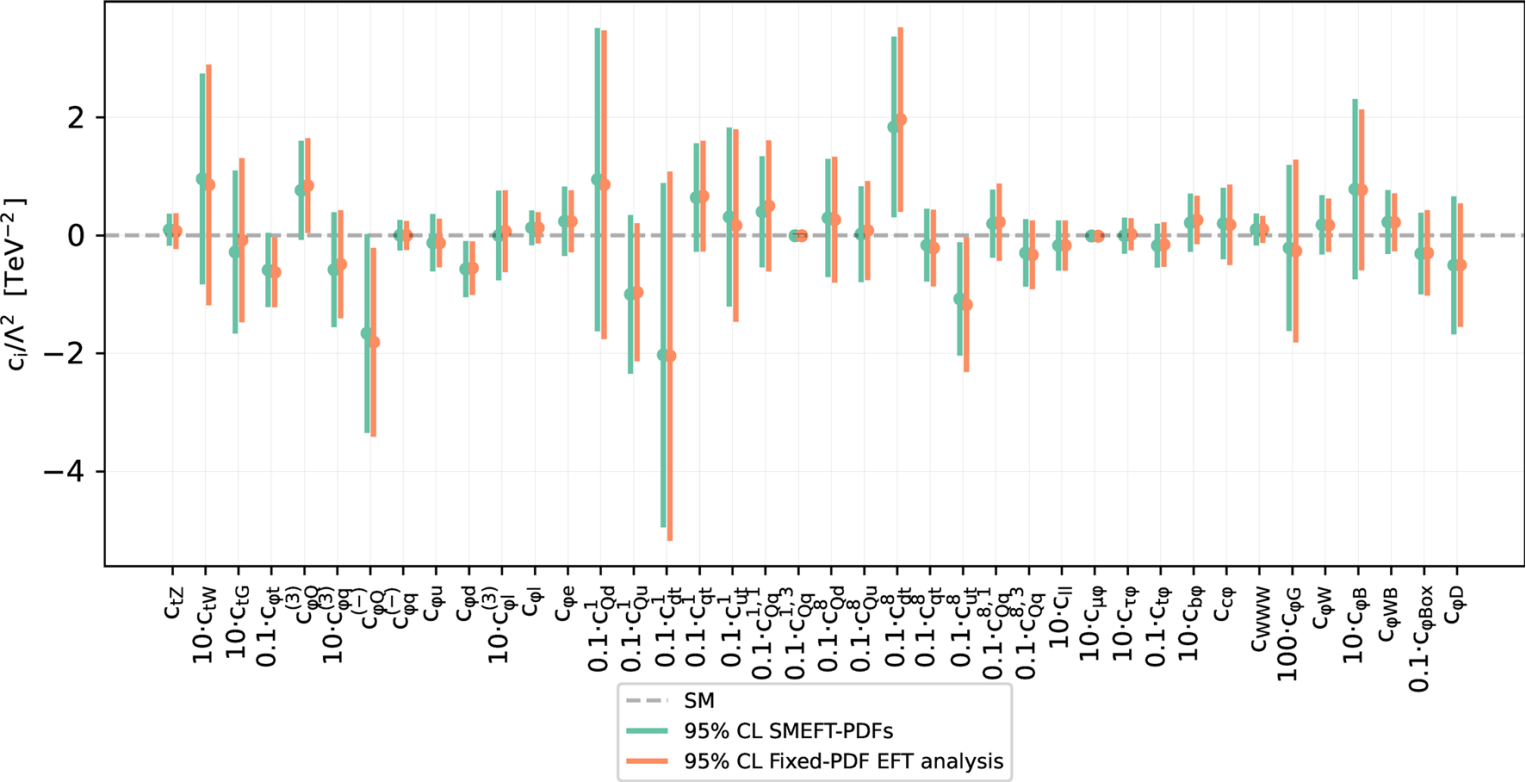
Comparison of the 95% CL intervals on the 40 Wilson coefficients considered in this work (in the linear EFT case) between the outcome of the joint SMEFT-PDF determination and that of the fixed-PDF EFT analysis. The latter is based on SM and EFT calculations performed with NNPDF4.0-notop as input. In both cases, results are based on the global dataset being considered and SMEFT cross-sections are evaluated up to linear, corrections. The dashed horizontal line indicates the SM prediction, $c_k = 0$. Note that some coefficients are multiplied by the indicated pre-factor to facilitate the visualisation of the results

**Fig. 11:**
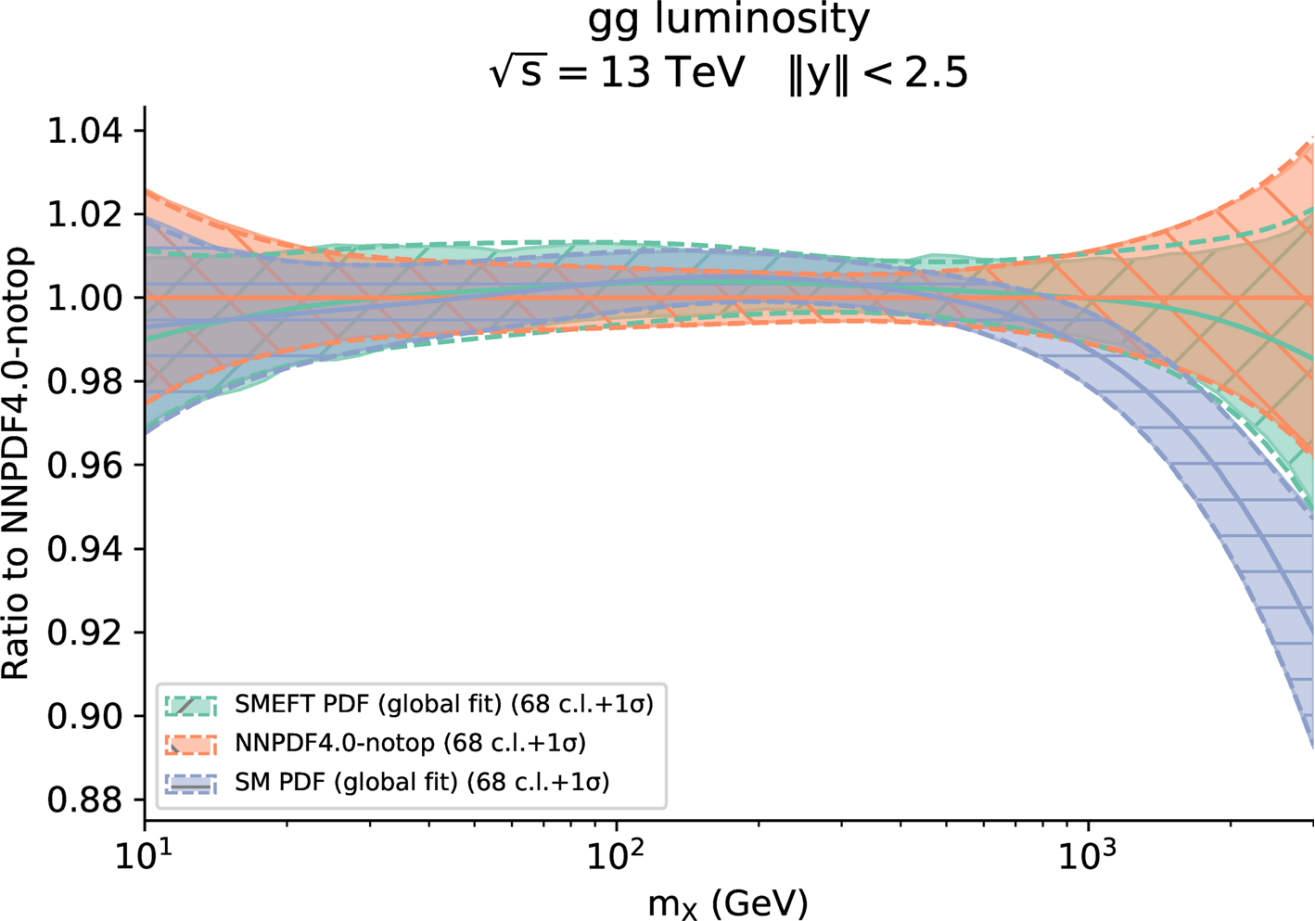
The gluon-gluon partonic luminosities at $\sqrt{s} = 13$ TeV as a function of the final-state invariant mass $m_X$. We compare the NNPDF4.0-notop baseline fit with its SM-PDF counterpart including all new data presented in Sect. 4.1, as well as with the simultaneous SMEFT-PDF determination. Results are presented as the ratio to the central value of the NNPDF4.0-notop baseline

Figure 9 displays a selection of the largest correlations. We observe that the gluon PDF in the medium to large-*x* region is significantly correlated with the Wilson coefficients $c_{tZ}$, as one would expect given that $c_{tZ}$ is strongly constrained by the top loop contribution to the Higgs decay, which in turn is predominantly correlated to the gluon-gluon parton luminosity. On the other hand $c_{\phi t}$ is anti-correlated with the gluon and correlated to the singlet and triplet in the large-*x* region. This is not surprising, given the impact of top quark pair production total cross sections and differential distributions in constraining these PDFs and Wilson coefficients. Whilst these correlations are computed from a determination of the SMEFT in which the PDFs are fixed to SM PDFs, the emergence of non-zero correlations provides an indication of the potential for interplay between the PDFs and the SMEFT coefficients; this interplay will be investigated in a simultaneous determination in the following section.

**Table 5:** The values of the $\chi ^2$ per data point for the $t{\bar{t}}$ production datasets that enter both the SMEFT-only fit and the simultaneous SMEFT-PDF fit. For each dataset, we indicate the number of data points $n_{\textrm{dat}}$ and the $\chi ^2$ for the SMEFT-only fit with PDF fixed to the input PDF NNPDF4.0-notop and for the simultaneous SMEFT-PDF determination. In bold we indicate the figures relative to the whole set of inclusive $t\bar{t}$ measurements

Dataset	$n_{\textrm{dat}}$	$\chi ^2/n_{\textrm{dat}}$	
		SMEFT-PDF fit	SMEFT fit
ATLAS $\sigma (t{\bar{t}})$, dilepton, 7 TeV	1	1.87	1.84
ATLAS $\sigma (t{\bar{t}})$, dilepton, 8 TeV	1	0.33	0.35
ATLAS $1/\sigma d\sigma /dm_{t{\bar{t}}}$, dilepton, 8 TeV	5	0.30	0.30
ATLAS $\sigma (t{\bar{t}})$, $\ell +$jets, 8 TeV	1	3$\cdot 10^{-5}$	3$\cdot 10^{-4}$
ATLAS $1/\sigma d\sigma /d|y_t|$, $\ell +$jets, 8 TeV	4	1.27	1.22
ATLAS $1/\sigma d\sigma /d|y_{t{\bar{t}}}|$, $\ell +$jets, 8 TeV	4	2.8	2.95
ATLAS $\sigma (t{\bar{t}})$, dilepton, 13 TeV	1	2$\cdot 10^{-3}$	2$\cdot 10^{-4}$
ATLAS $\sigma (t{\bar{t}})$, hadronic, 13 TeV	1	0.09	0.09
ATLAS $1/\sigma d^2\sigma /d|y_{t{\bar{t}}}|dm_{t{\bar{t}}}$, hadronic, 13 TeV	10	1.84	1.88
ATLAS $\sigma (t{\bar{t}})$, $\ell +$jets, 13 TeV	1	0.01	0.01
ATLAS $1/\sigma d\sigma /dm_{t{\bar{t}}}$, $\ell +$jets, 13 TeV	8	2.07	2.1
CMS $\sigma (t{\bar{t}})$, combined, 5 TeV	1	0.23	0.22
CMS $\sigma (t{\bar{t}})$, combined, 7 TeV	1	0.01	0.01
CMS $\sigma (t{\bar{t}})$, combined, 8 TeV	1	0.12	0.13
CMS $1/\sigma d^2\sigma /d|y_{t{\bar{t}}}|dm_{t{\bar{t}}}$, dilepton, 8 TeV	16	0.51	0.53
CMS $1/\sigma d\sigma /d|y_{t{\bar{t}}}|$, $\ell +$jets, 8 TeV	9	0.93	0.94
CMS $\sigma (t{\bar{t}})$, dilepton, 13 TeV	1	0.60	0.62
CMS $1/\sigma d\sigma /dm_{t{\bar{t}}}$, dilepton, 13 TeV	5	2.26	2.24
CMS $\sigma (t{\bar{t}})$, $\ell +$jets, 13 TeV	1	1.90	1.98
CMS $1/\sigma d\sigma /dm_{t{\bar{t}}}$, $\ell +$jets, 13 TeV	14	0.93	0.94
ATLAS charge asymmetry, 8 TeV	1	0.63	0.63
ATLAS charge asymmetry, 13 TeV	5	0.87	0.91
CMS charge asymmetry, 8 TeV	3	0.06	0.06
CMS charge asymmetry, 13 TeV	3	0.39	0.36
ATLAS & CMS combined charge asy., 8 TeV	6	0.60	0.61
ATLAS *W*-hel., 13 TeV	2	7$\cdot 10^{-5}$	1$\cdot 10^{-3}$
ATLAS & CMS combined *W*-hel., 8 TeV	2	0.33	0.34
Total inclusive $t{\bar{t}}$	**108**	**1.01**	**1.03**

**Table 6:** Same as Table 5 for associate $t{\bar{t}}$ and vector boson production

Dataset	$n_{\textrm{dat}}$	$\chi ^2/n_{\textrm{dat}}$	
		SMEFT-PDF fit	SMEFT fit
ATLAS $\sigma (t{\bar{t}}Z)$, 8 TeV	1	1.40	1.33
ATLAS $\sigma (t{\bar{t}}W)$, 8 TeV	1	0.62	0.71
ATLAS $\sigma (t{\bar{t}}Z)$, 13 TeV	1	2$\cdot 10^{-6}$	5$\cdot 10^{-3}$
ATLAS $1/\sigma d\sigma (t{\bar{t}}Z)/dp_T^Z$, 13 TeV	5	1.85	1.84
ATLAS $\sigma (t{\bar{t}}W)$, 13 TeV	1	2$\cdot 10^{-2}$	4$\cdot 10^{-3}$
ATLAS $\sigma (t{\bar{t}}\gamma )$, 8 TeV	1	0.33	0.29
CMS $\sigma (t{\bar{t}}Z)$, 8 TeV	1	2$\cdot 10^{-3}$	5$\cdot 10^{-3}$
CMS $\sigma (t{\bar{t}}W)$, 8 TeV	1	0.69	0.78
CMS $\sigma (t{\bar{t}}Z)$, 13 TeV	1	0.10	0.15
CMS $1/\sigma d\sigma (t{\bar{t}}Z)/dp_T^Z$, 13 TeV	3	0.86	0.88
CMS $\sigma (t{\bar{t}}W)$, 13 TeV	1	0.48	0.35
CMS $\sigma (t{\bar{t}}\gamma )$, 8 TeV	1	0.01	7$\cdot 10^{-3}$
**Total associated** $t{\bar{t}}$	**18**	**0.86**	**0.86**

**Table 7:** Same as Table 5, now for inclusive and associated single top datasets

Dataset	$n_{\textrm{dat}}$	$\chi ^2/n_{\textrm{dat}}$	
		SMEFT-PDF fit	SMEFT fit
ATLAS *t*-channel $\sigma (t)$, 7 TeV	1	0.24	0.26
ATLAS *t*-channel $\sigma ({\bar{t}})$, 7 TeV	1	0.01	0.02
ATLAS *t*-channel $1/\sigma d\sigma (tq)/dy_t$, 7 TeV	3	0.9	0.89
ATLAS *t*-channel $1/\sigma d\sigma ({\bar{t}}q)/dy_{{\bar{t}}}$, 7 TeV	3	0.06	0.06
ATLAS *t*-channel $\sigma (t)$, 8 TeV	1	0.01	0.02
ATLAS *t*-channel $\sigma ({\bar{t}})$, 8 TeV	1	0.30	0.24
ATLAS *t*-channel $1/\sigma d\sigma (tq)/dy_t$, 8 TeV	3	0.28	0.28
ATLAS *t*-channel $1/\sigma d\sigma ({\bar{t}}q)/dy_{{\bar{t}}}$, 8 TeV	3	0.19	0.20
ATLAS *s*-channel $\sigma (t + {\bar{t}})$, 8 TeV	1	0.02	0.04
ATLAS *t*-channel $\sigma (t)$, 13 TeV	1	0.31	0.32
ATLAS *t*-channel $\sigma ({\bar{t}})$, 13 TeV	1	0.05	0.03
ATLAS *s*-channel $\sigma (t + {\bar{t}})$, 13 TeV	1	0.05	0.03
CMS *t*-channel $\sigma (t) + \sigma ({\bar{t}})$, 7 TeV	1	0.02	0.02
CMS *t*-channel $\sigma (t)$, 8 TeV	1	0.34	0.31
CMS *t*-channel $\sigma ({\bar{t}})$, 8 TeV	1	0.98	1.07
CMS *s*-channel $\sigma (t + {\bar{t}})$, 8 TeV	1	1.42	1.45
CMS *t*-channel $\sigma (t)$, 13 TeV	1	0.34	0.35
CMS *t*-channel $\sigma ({\bar{t}})$, 13 TeV	1	0.02	0.03
CMS *t*-channel $1/\sigma d\sigma /d|y^{(t)}|$, 13 TeV	4	0.40	0.40
**Total inclusive single top**	**30**	**0.33**	**0.34**
ATLAS $\sigma (tW)$, dilepton, 8 TeV	1	0.07	0.05
ATLAS $\sigma (tW)$, single-lepton, 8 TeV	1	0.35	0.32
ATLAS $\sigma (tW)$, dilepton, 13 TeV	1	0.74	0.71
ATLAS $\sigma _{\text {fid}}(tZj)$, dilepton, 13 TeV	1	0.30	0.24
CMS $\sigma (tW)$, dilepton, 8 TeV	1	0.08	0.07
CMS $\sigma (tW)$, dilepton, 13 TeV	1	2.21	2.41
CMS $\sigma _{\text {fid}}(tZj)$, dilepton, 13 TeV	1	0.13	0.17
CMS $d\sigma _{\text {fid}}(tZj)/dp_T^{t}$, dilepton, 13 TeV	3	0.13	0.14
CMS $\sigma (tW)$, single-lepton, 13 TeV	1	1.51	1.42
**Total associated single top**	**11**	**0.53**	**0.53**

**Table 8:** Same as Table 5, now for the Higgs, diboson and electroweak precision observables. In bold we indicate the figures relative to the whole set of Higgs measurements, diboson measurements, EWPO measurements and of all the sets indicated in Tables 5–8

Dataset	$n_{\textrm{dat}}$	$\chi ^2/n_{\textrm{dat}}$	
		SMEFT-PDF fit	SMEFT fit
ATLAS STXS combination, $\mu _{H}$, 13 TeV	25	0.49	0.50
ATLAS signal strength $H \rightarrow Z \gamma$, 13 TeV	1	4$\cdot 10^{-5}$	3$\cdot 10^{-3}$
ATLAS signal strength $H \rightarrow \mu ^{+} \mu ^{-}$, 13 TeV	1	7$\cdot 10^{-3}$	2$\cdot 10^{-3}$
CMS signal strength combination, $\mu _{H}$, 13 TeV	24	0.66	0.67
ATLAS & CMS signal strength combination, $\mu _{H}$, 7 and 8 TeV	22	0.98	0.96
**Total Higgs**	**73**	**0.68**	**0.68**
ATLAS $d \sigma _{W^+W^-}/d m_{e \mu }$, 13 TeV	13	1.69	1.70
ATLAS $d \sigma _{WZ} / d m_{T}$, 13 TeV	6	0.77	0.77
ATLAS $d \sigma (Zjj)/d \Delta \phi _{jj}$, 13 TeV	12	0.79	0.79
CMS $d \sigma _{WZ} / d p_{T}$, 13 TeV	11	1.42	1.43
LEP $d \sigma _{WW} / d cos(\theta _W)$, 0.182 TeV	10	1.39	1.39
LEP $d \sigma _{WW} / d cos(\theta _W)$, 0.189 TeV	10	0.91	0.92
LEP $d \sigma _{WW} / d cos(\theta _W)$, 0.198 TeV	10	1.58	1.57
LEP $d \sigma _{WW} / d cos(\theta _W)$, 0.206 TeV	10	1.07	1.08
**Total Diboson**	**82**	**1.23**	**1.24**
LEP *Z* observables, 0.25 TeV	19	0.52	0.52
LEP ${\mathcal {B}}(W \rightarrow l^{-} {\bar{v}}_l)$ 0.196 TeV	3	2.59	2.59
LEP $\sigma (e^+ e^- \rightarrow e^+ e^-)$ 0.189 TeV	21	1.02	1.03
LEP $\hat{\alpha }^{(5)}(M_Z)$ 0.209 TeV	1	0.47	0.25
**Total EWPO**	**44**	**0.90**	**0.90**
**Total**	**366**	**0.91**	**0.91**

### Global simultaneous SMEFT and PDF fit

In this section we present the results of the simultaneous global fit of SMEFT Wilson coefficients and PDFs. We compare the bounds on the coefficients obtained in the two analyses as well as the resulting PDF sets, to assess whether the inclusion of more PDF-independent observables modifies the interplay observed in the top-focussed analysis of Ref. [21].

The first observation is that, similarly to the top-sector results observed in Ref. [21], in the global fit presented here the interplay between PDFs and SMEFT Wilson coefficients is weak. Indeed in Fig. 10 we observe that, including all data listed in Sect. 4.1, the bounds on the SMEFT Wilson coefficients are essentially identical in a SMEFT-only fit and in a simultaneous fit of SMEFT WCs and PDFs. The only mild sign of interplay is shown by the operator $c_{\phi t}$, which undergoes a fair broadening in the simultaneous fit compared to the fixed-PDF SMEFT fit.

The PDF fit is more interesting. It was shown in Ref. [21] that when comparing: (i) a SM PDF fit excluding top data, (ii) a simultaneous PDF-SMEFT using top data, (iii) a SM PDF fit including top data, there is a hierarchy of shifts in the gluon-gluon luminosity at high invariant masses. In particular, the gluon-gluon luminosity of the simultaneous fit (ii) is reduced at high invariant masses compared to fit (i), whilst the gluon-gluon luminosity of the SM fit including all top data (iii) is even further reduced at high invariant masses relative to the result of the simultaneous fit (ii). This can be explained due to the additional SMEFT degrees of freedom in (ii) allowing for a better description of the top data with the PDF remaining compatible with the no-top PDF.

As it can be observed in Fig. 11, in the new global fit presented in this paper, there is a shift in the gluon-gluon luminosity in the simultaneous PDF and SMEFT fit relative to the baseline no-top PDF set, however the shift is much smaller than the shift that one has by including all data in the SM, meaning that the effect of the inclusion of the top, Higgs, EWPO and diboson data has a much smaller effect on the PDFs due to the interplay between the pull of the SMEFT coefficients and the pull of the new data on the PDFs. The shift is even smaller compared to the shift of the simultaneous top fit relative to the no-top PDF presented in Ref. [21]. This is due to the inclusion of new high-mass Drell–Yan data in the new fit, which were not included in NNPDF4.0. These data favour a softer singlet PDF combination in the high-mass region, and as a result they enhance the gluon in the same region, due to sum rules.

Finally, it is also interesting to compare the fit quality of the SMEFT fit keeping PDF fixed and the simultaneous SMEFT-PDF fit. We display the $\chi ^2$ per datapoint in Tables 5, 6,  7 and  8 for each dataset appearing in both the simultaneous SMEFT-PDF fit and the fixed PDF SMEFT fit. The fit quality for inclusive and associated top pair production is shown in Table 5, for inclusive and associated single top production in Table 7 and for Higgs, diboson and electroweak precision observables in Table 8. We also display the total fit quality of the 366 SMEFT-affected datapoints in Table 8.

Overall, we observe that the fit quality of the total dataset remains stable between the SMEFT-PDF fit and the SMEFT fit, with a total $\chi ^2$ of 0.91 for both fits, as shown in Table 8. This is also observed in the Higgs and electroweak precision observables, while in the diboson sector a small improvement from $\chi ^2 = 1.24$ to $\chi ^2 = 1.23$ is observed. A similar improvement in the $\chi ^2$ is observed in the inclusive top quark pair production datasets in Tables 5 from $\chi ^2=1.03$ to $\chi ^2=1.01$, and in inclusive single top datasets in Tables 7 from $\chi ^2=0.34$ to $\chi ^2=0.33$, indicating that in these sectors, the SMEFT-PDF fit provides a better fit to the data. This improvement in the $\chi ^2$ in these sectors is not as significant as the improvement observed in Ref. [21], however. As discussed below Fig. 11, this is due to the inclusion of new high-mass Drell–Yan data in the new fit.

## Usage and applicability of the code

In this section we define the realm of applicability of SIMUnet and summarise a series of caveats that users should be aware of when using our tool for phenomenological applications.

- **Truncation of the SMEFT expansion:** In a simultaneous fit of PDF and SMEFT Wilson coefficients, SIMUnet only includes the linear SMEFT correction terms of $\mathcal{O}(1/\Lambda ^2)$, i.e. the interference between the SM and the SMEFT amplitudes. The reason why a simultaneous fit of PDF and SMEFT Wilson coefficients including quadratic SMEFT corrections based on the Monte Carlo replica sampling method would not give faithful confidence intervals for the SMEFT coefficients is explained in details in Ref. [82]. There it is shown that the uncertainty estimates of the fitted parameters are discrepant between the Monte Carlo replica method and a numerical Bayesian method (like for example a nested sampling approach) beyond linear models. On the other hand, in the context of ’contaminated’ fits there is no restriction to linearity, as pseudodata can be produced with any BSM model correction (EFT or UV complete). In this case only the PDFs are fitted on pseudo-data that are generated according to a given BSM model, and linearity around the best-fit of PDF parameters is assumed to be a good approximation independently of the linearity of the underlying BSM model.
- **Analysing flat directions: ** Given a selection of data to include in a fit, it is important to choose the Wilson coefficients to fit such that there are no unconstrained parameters or directions in the fit. It is possible to do this using the principal component analysis (PCA) function available in the simunet_analysis.py set of analysis tools. The PCA proceeds as follows. The covariance between Wilson coefficients $c_i$, $c_j$ is described by the covariance matrix $U_{ij} = (F^{-1})_{ij}$, where *F* denotes the Fisher information matrix given in Eq. (4.1). The Fisher information matrix $F_{ij}$ depends only on the theory predictions *T*(*c*) and on the experimental covariance matrix $\Sigma _{\textrm{exp}}$, and not on the values of the Wilson coefficients, and can therefore be computed pre-fit assuming a fixed input PDF set. The PCA consists of computing the eigenvalues of $F_{ij}$ using a singular value decomposition. If all eigenvalues are nonzero, we can conclude that *F* is an invertible matrix and $U_{ij}$ can be computed, implying that all coefficients in the fit will be constrained. On the other hand, if there are eigenvalues equal to zero, the combination of parameters leading to these unconstrained directions is indicated by the eigenvectors: these parameters can then be removed from the fit.
- **Correlation between PDFs and SMEFT corrections in the** *K***-factors:** The SMEFT *K*-factors defined in Eq. (2.8) are determined with a fixed choice of reference PDF, given that $\pmb {\omega } = \pmb {\omega }^*$, where they do actually depend on PDFs freely. In practice however, this approximation is often justified, as the PDF dependence cancels out to a good approximation. To verify the level of accuracy of such approximation, that consists in neglecting the correlation at the pre-fit level between the SMEFT corrections and the PDFs, one can recalculate the *K*-factors post-fit, by using as input PDF set the one coming out of the simultaneous fit and verify that the *K*-factors are stable, otherwise the user should recompute them recursively until stability is reached, as it is discussed in App. C of Ref. [33].
- **Number of replicas: ** Throughout this study we use fits of $N_{\text {rep}} = 1000$ replicas, both in fixed-PDF and in simultaneous fits. This ensures that central values, covariance and correlations of experiments are reproduced to a good accuracy at 1% level for all experiments. Still, it has been shown that even $N_{\text {rep}} = 100$ replicas is sufficient to obtain a good estimate of the solutions in the case of relatively few Wilson coefficients, and adding more replicas causes little change [35]. If possible, in terms of computational resources, using $N_{\text {rep}} = 1000$ is the standard to ensure that the results are stable and robust. Increasing the number of Wilson coefficients in the fit amounts to simply having some more trainable weights in the SIMUnet architecture, and does not really affect the number of replicas required.
- **Choice of scale parameter: ** Choosing appropriate scale parameters is important for the optimal performance of the SIMUnet framework, in the same way that choosing correct learning rates is important for any NN architecture. As described in Sect. 2.3, choosing a scale parameter $\lambda$ to fit a Wilson coefficient *c* amounts to multiplying the associated learning rate by $\frac{1}{\lambda }$, or equivalently, fitting $\lambda c$ instead of *c*. In practice, choosing a small scale gives large steps during the gradient descent which can speed up the convergence but at the risk to probe regions of the parameter space where the $\chi ^2$ is gigantic. This can also create oscillatory-like behaviours toward the end of the fit if the minimum is found for a value of *c* much smaller than the step. On the other hand, choosing a large scale reduces the risk of probing regions of the parameter space very far from the minimum, provided the initialisation conditions are reasonable. It also reduces the risk of oscillation around the minimum. However, it may slow down the convergence if the fit is initialised far away from the minimum and, in the worst case scenario, it may prevent the fit from converging before the maximum number of allowed iterations is reached. It is important to note that, usually, the optimal scale choice for each Wilson coefficient is found a posteriori. In practice, it is useful to first launch an exploratory SMEFT-only fit to obtain a preliminary estimate of the size of the bounds and central values of the Wilson coefficients, and then use this information to set the scale parameters, which are usually of ${\mathcal {O}}(10)$ or smaller, so that the Wilson coefficients is of ${\mathcal {O}}(1)$. Naturally, it is expected that PDFs and SMEFT coefficients will change in the case of fixed-PDF or simultaneous fits. Depending on the dataset, the overall fit quality may change very little, but the PDFs can accommodate better the inclusion of new data. In this way, the impact of the new data on the PDFs is diluted, and the constraining power of the global dataset is also absorbed by the Wilson coefficients.

## Conclusions

In this work we have presented the public release, as an open-source code, of the software framework underlying the SIMUnet global simultaneous determination of PDFs and SMEFT coefficients. Based on the first tagged version of the public NNPDF code [38, 39], SIMUnet adds a number of new features that allow the exploration of the interplay between a fit of PDFs and BSM degrees of freedom.

The SIMUnet code is publicly available from its GitHub repository:

https://github.com/HEP-PBSP/SIMUnet,

and is accompanied by documentation and tutorials provided at:

https://hep-pbsp.github.io/SIMUnet/.

We have documented the code and shown the phenomenological studies that can be performed with it, that include (i) a PDF-only fit *à la* NNPDF4.0 with extra datasets included; (ii) a global EFT analysis of the full top quark sector together with the Higgs production and decay rates data from the LHC, and the precision electroweak and diboson measurements from LEP and the LHC; (iii) a simultaneous PDF-SMEFT fit in which the user can decide which observable to treat as PDF-dependent or independent by either fitting PDFs alongside the Wilson coefficients or freezing them to a baseline PDF set.

We have demonstrated that the user can use the code to inject any New Physics model in the data and check whether the model can be fitted away by the PDF parametrisation. We have also shown that the methodology successfully passes the closure test with respect to an underlying PDF law and UV “true” model that is injected in the data.

The new result presented here is the global analysis of the SMEFT and explicitly shows that SIMUnet can be used to produce fits combining the Higgs, top, diboson and electroweak sectors such as those in Refs. [25–27], and that more data can be added in a rather straightforward way to combine the sectors presented here alongside Drell–Yan and flavour observables, as it is done in Ref. [31]. We have presented the results and compared them to existing fits. We have shown that the interplay between PDFs and SMEFT coefficients, once they are fitted simultaneously, has little effect on the Wilson coefficients, while it has the effect of shifting the gluon–gluon luminosity and increasing its uncertainty in the large-$M_X$ region.

The public release of SIMUnet represents a first crucial step towards the interpretation of indirect searches under a unified framework. We can assess the impact of new datasets not only on the PDFs, but now on the couplings of an EFT expansion, or on any other physical parameter. Indeed, the methodology presented here can be extended to simultaneous fitting precision SM parameters, such as the strong coupling or electroweak parameters. Indeed, our framework extends naturally, not only to BSM studies, but to any parameter which may modify the SM prediction through the use of K-factors or other kind of interpolation, see Appendix A of Ref. [35] for the application of SIMUnet to the simultaneous fit of PDFs and $\alpha _s(M_z)$ [83].

## Data Availability

My manuscript has associated data in a data repository. [Author’s comment: The datasets generated during and/or analysed during the current study are available in the SIMUnet and NNPDF repositories at https://github.com/HEP-PBSP/SIMUnet and https://github.com/NNPDF respectively.]

## Footnotes

1 Technically, the form $f_i(x,Q_0^2) = x^{\alpha _i} (1-x)^{\beta _i} NN_{i}(x,\pmb {\omega })$ is used for each of the fitted PDF flavours, i.e. a power law scaling is used as a pre-factor; we will ignore this to streamline the presentation. Indeed, it was recently shown in Ref. [44] that this pre-factor can be removed completely, and recovered using only the neural network parametrisation as presented in this text.

2 The full NNPDFcode documentation is provided at https://docs.nnpdf.science.

3 Note that in Sect. 5.3 of Ref. [35], it was initially proposed that SIMUnet: would operate in *non-linear* regimes too. However, due to the findings regarding uncertainty propagation using the Monte Carlo replica method presented in App. E of Ref. [21], the non-linear feature has been deprecated in this public release of the code.

4 Recall that if ${\textbf {v}} = {\textbf {w}} \odot {\textbf {z}}$, the elements of ${\textbf {v}}$ are defined by $v_i = w_i z_i$.

5 See the documentation in https://hep-pbsp.github.io/SIMUnet/ for a list of available fits to preload.

6 Assuming that the PDFs can be parametrised by a neural network, and then subsequently using a neural network to perform the fit, ignores modelling error; we do not consider this here for simplicity, but both the NNPDF4.0 and SIMUnet frameworks can account for it.

7 In practice, an ensemble of fits is produced, and if their 68% confidence bands cover the true law in 68% of the fits, we say that the methodology has passed the closure test.

## Code Availability Statement

My manuscript has associated code/software in a data repository. [Author’s comment: The code/software generated during and/or analysed during the current study is available in the SIMUnet repository at https://github.com/HEP-PBSP/SIMUnet.]
